# A bottom-up approach to construct or deconstruct a fluid instability

**DOI:** 10.1038/s41598-021-03676-z

**Published:** 2021-12-21

**Authors:** Darío M. Escala, Alberto P. Muñuzuri

**Affiliations:** Group of Nonlinear Physics, Department of Physics, University of Santiago de Compostela, 15782 Santiago de Compostela, Spain

**Keywords:** Fluid dynamics, Nonlinear phenomena, Chemical physics, Surfaces, interfaces and thin films

## Abstract

Fluid instabilities have been the subject of study for a long time. Despite all the extensive knowledge, they still constitute a serious challenge for many industrial applications. Here, we experimentally consider an interface between two fluids with different viscosities and analyze their relative displacement. We designed the contents of each fluid in such a way that a chemical reaction takes place at the interface and use this reaction to suppress or induce a fingering instability at will. This process describes a road map to control viscous fingering instabilities in more complex systems via interfacial chemical reactions.

## Introduction

Fluid instabilities at the interface between two fluids are ubiquitous in nature and are responsible for important phenomena that affect everybody’s life^1,2^. In some cases, they play a constructive role like in the redistribution of energy in a system but in some other cases, the role is destructive and may pose a serious threat to technical or industrial installations. In most cases, these fluids involve reactants that are known to modify the instability itself^3–10^. Enhanced oil recovery techniques are a clear example where the role of this instability is crucial^11^. A great effort has been done in understanding these phenomena and just recently the effects of reactants have been considered.

We propose in this manuscript a bottom-up approach in order to control instabilities. We focus on instabilities originating at a viscosity jump between two fluids (typically viscous fingering). We develop a system that is likely to produce instabilities and we endow it with the appropriate chemical reactions at the interface that allow us to control the activation or deactivation of the instability at will. In particular, we consider two different fluids with different viscosities and analyze the displacement of one fluid by the other that is being injected into the system. Our results establish the basis to control fluid instabilities that may arise in a broad variety of contexts.

The manuscript is organized as follows. First, we consider the a priori stable configuration with a more viscous fluid displacing a less viscous one and induce an instability at the interface by means of the appropriate chemical reactions. The second part of the paper considers the reverse situation and the interfacial reaction is, then, used to suppress the instability. The final part of the results section is devoted to the full understanding of the mechanism involved and the mathematical model that describes it.

## Materials and methods

### Experimental section

The system is composed of two solutions, A and B, named displaced and displacing solutions depending on the case of study. One of the solutions, solution A, was prepared by mixing stocks of Poly(Acrylic Acid) (PAA), sodium sulfite, formaldehyde, and pH indicator as follows: The PAA solution was prepared by diluting 1 g of the reagent grade polyacrylic acid with an average molecular weight of 4,000,000 g mol^−1^ (Sigma), into 180 ml of doubly-distilled water at 80 °C to facilitate solubility. After complete solubilization, the mixture was cooled down to 23 °C and the final volume was kept at 200 ml obtaining a PAA stock solution of 0.5 wt%. The formaldehyde was used directly as a stock solution from a commercial formalin solution (Sigma-Aldrich). The sodium sulfite stock solution was prepared from reagent grade Na_2_SO_3_ (Sigma) diluting 25.21 g of the reagent in 100 mL of double distilled water bubbled with argon to avoid oxidation. We included in solution A a pH color indicator (hereafter C.I.) in order to visually monitor changes in pH. We considered as C.I. a 0.4 wt% hydroalcoholic solution of Bromothymol blue prepared by dissolving 1 g of Bromothymol blue sodium salt powder (Sigma) into 50 ml of a 96% ethanol solution diluting up to a final volume of 250 mL by adding 200 mL of doubly-distilled water. The C.I. shows a yellow color for pH values below 6 (acidic state), green color for pH between 6–7 (neutral state), and a blue color for pH values above 7 (basic state)^12^. The reagent concentrations in solution A used for all experiments are: PAA 0.438 wt%, 0.068 M SO_3_^2−^, 0.021 wt% C.I. and 0.350 M Formaldehyde. This solution has a blue coloration and a pH that is between 11.5 and 12. The other solution, solution B, is a concentrated aqueous solution of gluconic acid 2.0 mol/kg (≈1.66 M) obtained by diluting 17.81 g of D-(+)-gluconic acid δ-lactone (Sigma) into 50 g of doubly distilled water. The solution was left to rest a complete day to ensure the full conversion of the gluconolactone into gluconic acid by hydrolysis^13^.

A complete characterization of the rheological properties of the components of solution A and B have been previously reported^14^. Moreover, pattern formation in a radial injection framework involving similar solutions was also studied in Escala *et al*^8^. Both works show a complete characterization of the system evolution by varying not only the chemistry^14^ but also the interfacial interaction^8^.

**Figure 1:**
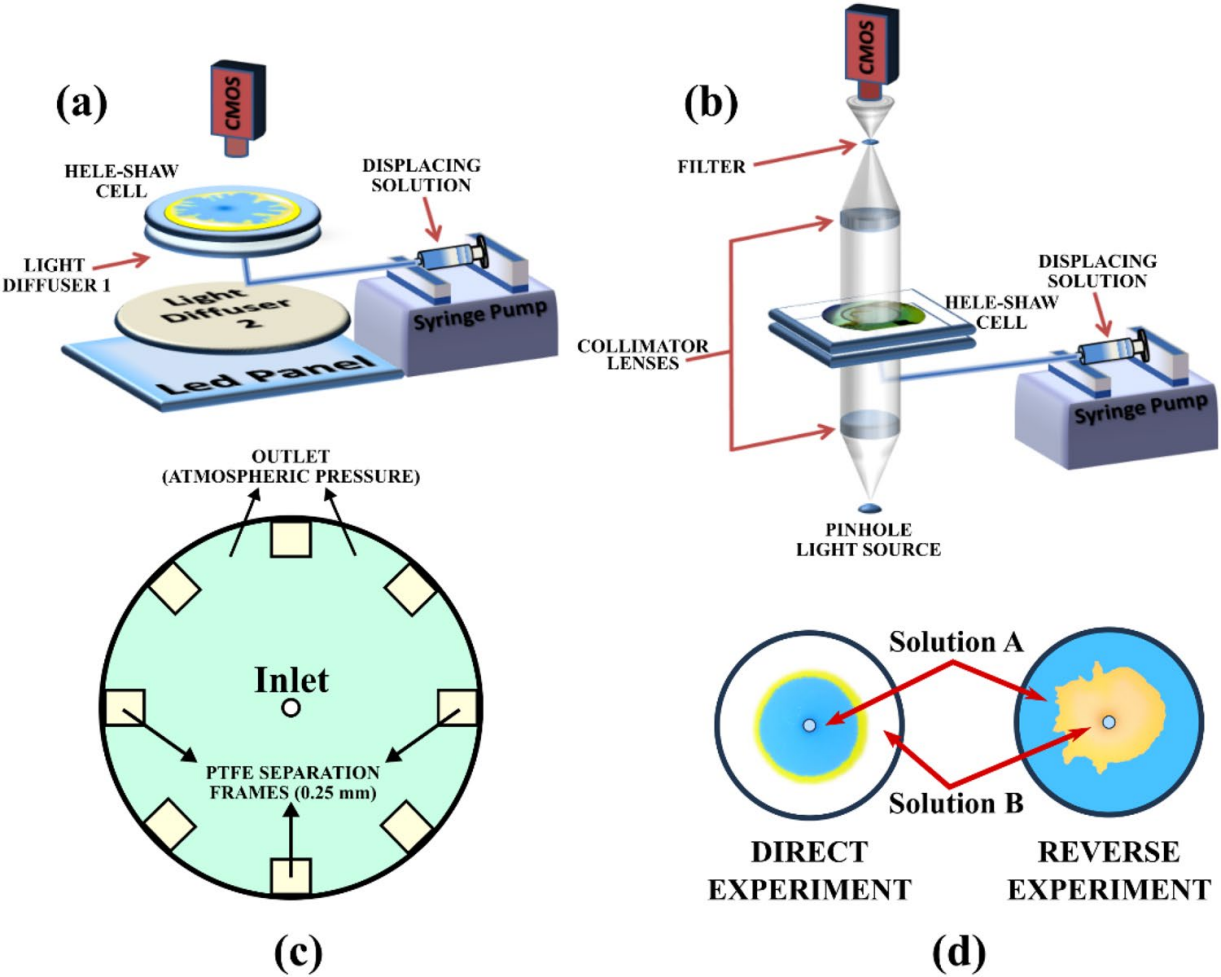
Schematics of the experimental setup. (**a**) Configuration for visible light detection (colored experiments). (**b**) Schlieren arrangement for optical detection of gradients of the index of refraction in the absence of color indicator. (**c**) Schematic of the configuration of Hele–Shaw cell outputs. The two parallel plates are separated by PTFE frames of 0.25 mm each located at the border of the cell. Each frame is separated permitting a homogenous flow distribution. The cell output is at atmospheric pressure. (**d**) Schematics of the configuration of the displacing-displaced fluid for each case studied.

The sketch of the experimental setup used is shown in Fig. 1 (similar to the one used in Escala *et al*^8^). The Hele–Shaw cell was built using two circular Poly(methyl methacrylate) plates (18 cm diameter) (Plexyglass®) separated by several Polytetrafluoroethylene (PTFE) frame of 0.25 mm of thickness each as indicated in Fig. 1c. The cell output is at atmospheric pressure permitting a homogeneous flow distribution along the radial coordinate.

The cell was initially filled with the displaced solution. The displacing solution was injected through a 4 mm hole located at the geometric center of the bottom plate using a syringe pump (kdScientific: Legato 200 series). Two cases are considered by switching between displaced and displacing solutions (Fig. 1d). In the so-called direct experiment, solution A and B are the displacing and displaced solutions respectively. In the reverse experiments, the opposite situation is considered (solution B is the displacing and solution A is the displaced solution).

Special care must be taken when injecting the less viscous fluid into the more viscous one (reverse experiments). The displacing fluid must be directly injected into the Hele–Shaw cell avoiding any contact between fluids in the injection tubes or connectors. The large viscosity difference may produce viscous fingering inside the tubes that would lead to undesired effects on the experiments.

Experiments are recorded from above using a Complementary Metal Oxide Semiconductor (CMOS) camera (PixeLINK: PL-B776U) connected to a computer. Samples were illuminated from below using a rectangular Light Emitting Diode (LED) pad and a light diffuser (Fig. 1a). The image post-processing was done with the GNU software FIJI^15^.

All experiments are done in a temperature-controlled environment. Solutions are thermally stabilized at 23 °C by using a thermostatic bath. Note that the light source used is an array of LEDs located at some distance from the cell, so its contribution to changes in the temperature is negligible.

#### Schlieren imaging

In order to track changes in the optical index induced by the motion of fluids that cannot be observed by a direct optical inspection, we used the Schlieren technique^16–18^. In our case, the Schlieren technique is also useful to appreciate changes in the polymer solution due to reactive effects and polymer aggregation^8^. For this experimental design (see Fig. 1b), the Hele–Shaw cell was made of two square glass plates (25 cm × 25) instead of the plastic plates. This modification was introduced to discard any artifact associated with the cell material and to improve the image quality. To perform the Schlieren measurements, the Hele–Shaw cell was placed between two collimator lenses. The system was illuminated using a pinhole LED light source. An iris cutoff filter was located at the focus point between the CMOS and the collimator lens as indicated in the scheme presented in Fig. 1b^8,18,19^.

#### Image analysis: circularity

**Figure 2:**
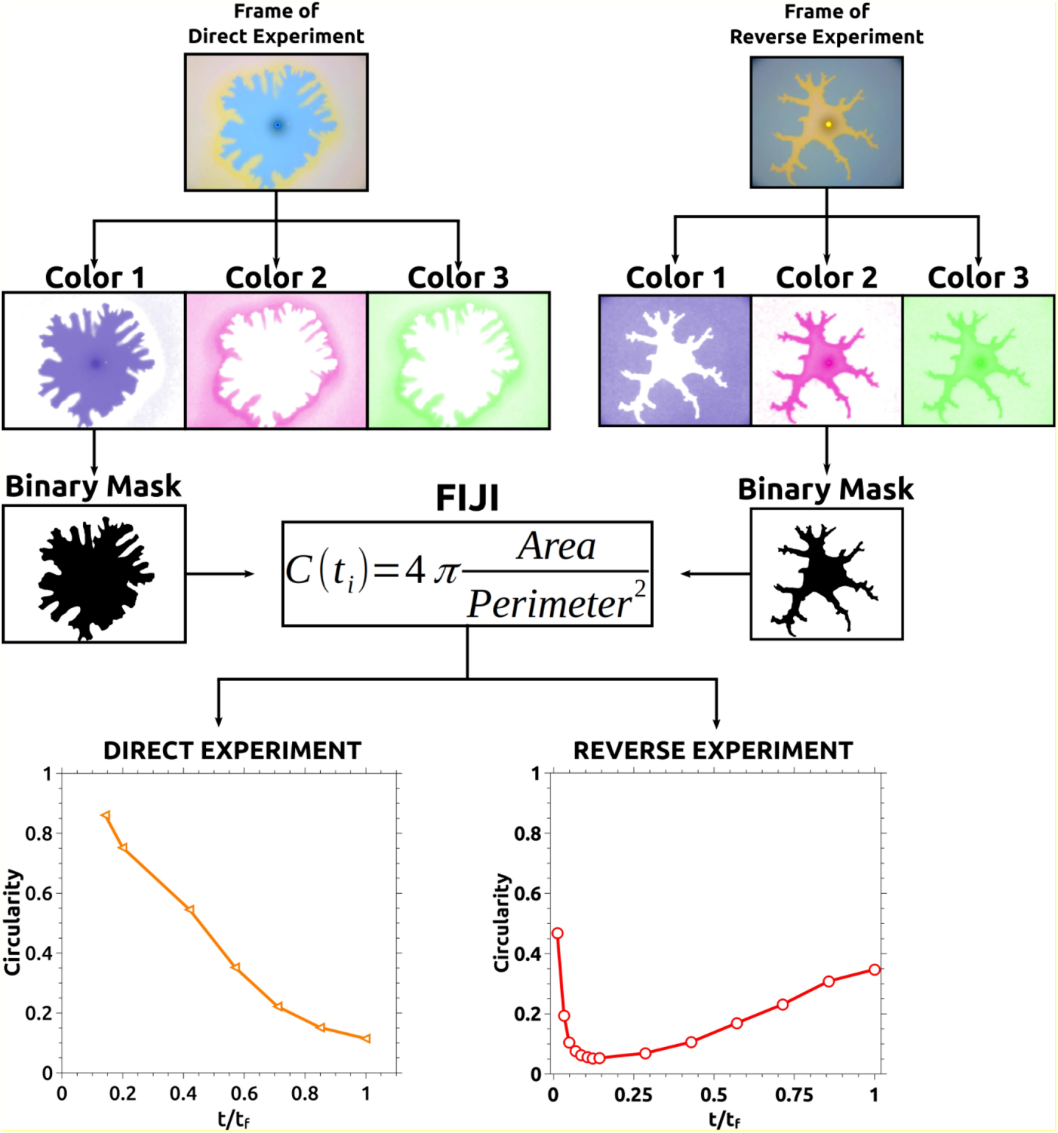
Methodology to calculate the circularity. The frames of every experimental case are processed with FIJI^15^ by using a color deconvolution algorithm. From such a process, three color components are obtained for each frame. Color 1 and 2 are used to obtain a binary mask for each type of experiment. From such a mask, the circularity is calculated as indicated in the scheme. A circularity value is obtained for every processed frame. Those values are then represented in a relative time scale for comparison.

To quantify the effect of the reaction in the obtained patterns, we measure a morphological descriptor called circularity defined as C = 4π[area/perimeter^2^]. This descriptor indicates how close a region of interest is to a perfect circle. The value of C is calculated directly using FIJI^15^. The methodology is presented in Fig. 2. Every analyzed frame of each studied case is processed by the Hematoxylin and Eosin (H&E) color deconvolution algorithm. Other algorithms included in the software give similar results, however, the default algorithm was the simplest choice. Once processed, three color components are obtained. A binary mask is then created from one of the color components. For the direct experiments, the best results are obtained binarizing the first color component (Color 1 in the schematics of Fig. 2). For the reverse experiments, the second color component is chosen (Color 2 in the schematics of Fig. 2). Once obtained the binary images, the software calculates the circularity following the definition. As several frames of a complete experimental run are processed, the evolution of the circularity can be plotted as a function of time.

#### Numerical setup

**Figure 3:**
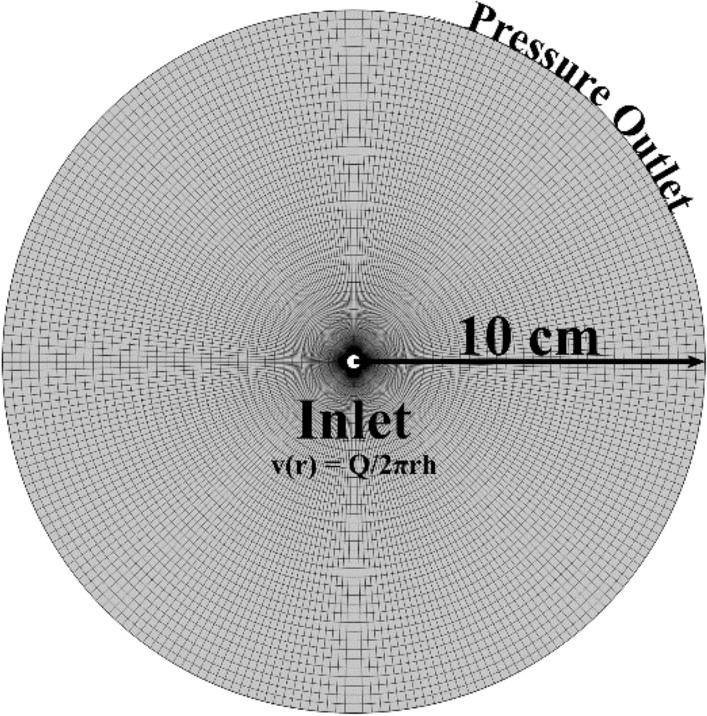
The 2D-RDA simulation domain is composed of a circular region of 10 cm radius discretized using a mapped mesh of radial elements. The inlet is located at the central inner hole and the velocity v_0_ is calculated from the flow rate Q. The outer boundary is set as a constant pressure condition with P = 0 Pa.

Simulations are performed using the computational fluid dynamic (CFD) software suite ANSYS Fluent 20.1^20^. The numerical domain is composed of a circular region of radius R = 10 cm discretized using a mapped mesh of radial elements (Fig. 3). The inlet flow velocity at the central boundary is calculated as

1 $$- \vec{n} \cdot \vec{u} = v_{0} \left( Q \right)$$

where $v_{0}$ = *Q/2πrh*. The injection hole is the circular region of radius r = 2 mm located at the geometric center of the domain. The outlet boundary condition was set as a pressure outlet with p = 0 Pa. The pressure and species fields are discretized using second-order and first-order upwind schemes, respectively. Simulations are performed using the SIMPLE algorithm with a variable time-step setting. The mesh size is chosen after performing a mesh independence study (results are included in the S.I.). The system is simulated using real units to facilitate the comparison with the experiments.

The initial conditions are set using the following piecewise functions depending on the experimental case:

2a $${\text{Direct}}\;{\text{Experiment:}}\left\{ {\begin{array}{*{20}l} {A\left( {\vec{r},0} \right) = \left\{ {\begin{array}{*{20}l} {A_{0} \left( {1 + \xi N\left( {\vec{r}} \right)} \right),} \hfill & {\vec{r} \le \varepsilon } \hfill \\ {0,} \hfill & {\vec{r} > \varepsilon } \hfill \\ \end{array} } \right.} \hfill \\ {B\left( {\vec{r},0} \right) = \left\{ {\begin{array}{*{20}l} {B_{0} \left( {1 + \xi N\left( {\vec{r}} \right)} \right),} \hfill & {\vec{r} \ge \varepsilon } \hfill \\ {0,} \hfill & {\vec{r} < \varepsilon } \hfill \\ \end{array} } \right.} \hfill \\ {C\left( {\vec{r},0} \right) = 0, \forall {\vec{\text{r}}}} \hfill \\ \end{array} } \right.$$

2b $${\text{Reverse}}\;{\text{Experiment:}}\left\{ {\begin{array}{*{20}l} {A\left( {\vec{r},0} \right) = \left\{ {\begin{array}{*{20}l} {0,} \hfill & {\vec{r} < \varepsilon } \hfill \\ {A_{0} \left( {1 + \xi N\left( {\vec{r}} \right)} \right),} \hfill & {\vec{r} \ge \varepsilon } \hfill \\ \end{array} } \right.} \hfill \\ {B\left( {\vec{r},0} \right) = \left\{ {\begin{array}{*{20}l} {0,} \hfill & {\vec{r} \ge \varepsilon } \hfill \\ {B_{0} \left( {1 + \xi N\left( {\vec{r}} \right)} \right),} \hfill & {\vec{r} < \varepsilon } \hfill \\ \end{array} } \right.} \hfill \\ {C\left( {\vec{r},0} \right) = 0, \forall {\vec{\text{r}}}} \hfill \\ \end{array} } \right.$$

where *N* ($\vec{r}$) is a normally distributed random noise function of amplitude ξ = 0.01 set across the radial coordinate, and ε = 3 mm sets the initial contact region between fluids A and B included for stability reasons. A_0_ and B_0_ are the initial concentrations of solutions A and B respectively. For simplicity, both values are set equal to 1 mol/L. Simulations are carried out up to a final time calculated as t_f_ = V_f_/Q, where V_f_ is the total volume of displacing solution injected and it is fixed for all simulations. The remaining simulations parameters are listed in Table 1.

**Table 1:** Parameters used in the Reaction–Diffusion–Advection (RDA) simulations.

Parameter	Value	Dimension Unit	Reference/notes
*A*_*0*_	1	mol L^**−**1^	
*B*_*0*_	1	mol L^**−**1^	
*C*_*0*_	0	mol L^**−**1^	
*C*_*m*_	1	mol L^**−**1^	
*µ*_*A*_	1	Pa s	Experimental/adjusted
*P*_*0*_	0	Pa	
*D*_*A*_	1.0 × 10^–11^	m^2^ s^**−**1^	Estimated, Ref^21–23^
*D*_*B*_	9.3 × 10^–9^/1.0 × 10^–9^	m^2^ s^**−**1^	Estimated, Ref^24,25^
*D*_*C*_	0	m^2^ s^**−**1^	
*h*	0.25	mm	Experimental
*R*	10	cm	Experimental
*r*	2	mm	Experimental
ϕ	1		
*κ*_*0*_	5.208 × 10^–9^	m^2^	Experimental, *h*^2^/12
*k*_*1*_	5.2 × 10^–4^	L (mol s)^**−**1^	Experimental/adjusted
*k*_*2*_	5.2 × 10^–5^	L (mol s)^**−**1^	*k*_*1*_/10
*R*_*κ*_	80		Adjusted
*R*_*b*_	− 7		Adjusted
*V*_*f*_	7000	µL	Experimental
*t*_*f*_	Varies	min	Calculated as V_f_/Q

The value of D_B_ changes depending on the reactivity of the system. A value of D_B_ = 1.0 × 10^–9^ m^2^/s is used for the non-reactive case.

## Results

### Direct experiment: viscous solution displaces a less viscous solution

We designed two different solutions with completely different viscosities as described in the Methods section. The large viscosity solution (solution A) contains a large molecular weight (4,000,000 g/mol) Poly(Acrylic Acid) (PAA). The rest of the components of both solutions are selected and distributed in such a way that, at the interface and upon reaction, a dramatic change in the pH takes place. Solution A contains sodium sulfite, formaldehyde, and a pH color indicator as well as PAA, while solution B only contains gluconic acid. In this way, solution A has a significantly larger viscosity^14^: µ_A_ = 150 mPa.s and µ_B_ = 2.2 mPa.s measured at a shear rate of γ_r_ = 50 s^**−**1^.

**Figure 4:**
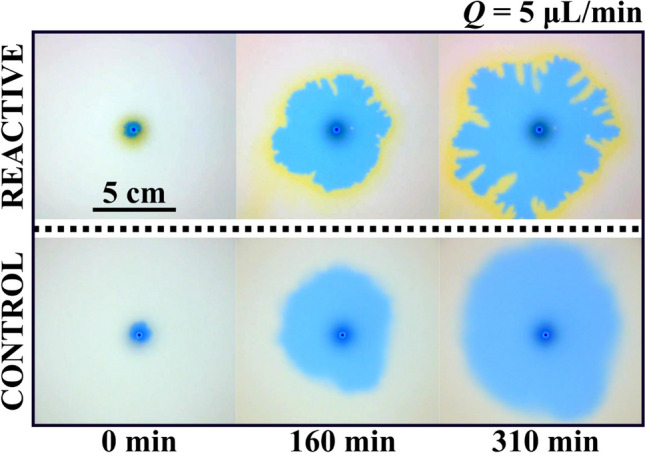
Comparison between a reactive (upper row) and non-reactive (lower row) case for a flow rate of 5 μL/min with the same experimental conditions. As observed, the radial profile of the displacing solution in the control case remains mostly circular as expected from a hydrodynamically stable configuration. Some disturbances are observed due to diffusive effects. In the reactive case, the displacing profile remains circular for the first minutes. Around t = 160 min, the circular shape deforms showing digitations. At t = 310 min, those ramifications enlarge resulting in a fully developed instability. The yellow zone next to the displaced fluid indicates the region where the reaction takes place. The displaced solution in the reactive case is gluconic acid while in the control case is H_2_O.

In fluid dynamics, the displacement of a fluid by a more viscous one is a stable configuration under any flow rate considered if no reactive processes are involved^3,7,10,26^. This phenomenon is observed in Fig. 4 (lower row labeled Control). Here, solution A (large viscosity) is pumped into the less viscous solution (for this control experiment, solution B is replaced by distilled water to avoid reaction) placed in advance inside a radial Hele–Shaw cell (see Methods for details). Three different snapshots are shown at different moments of the experiment. Note that in all pictures the geometry of the interface is circular, and no instability is observed. The interface becomes blurred as time goes on because both liquids are miscible. Note that in this control experiment, there is no interfacial chemical reaction.

**Figure 5:**
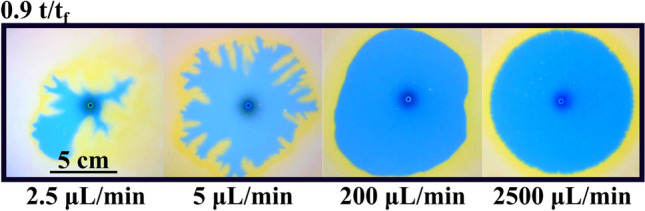
Effect of the flow rate as a trigger for the instability via a reactive interface. All the images are taken at 90% of the final time (t_f_) for each case. For Q = 2500 µL/min, the displacing interface remains completely circular. In this case, the characteristic flow time scale is much larger compared to the reaction time scale and a circular stable pattern is obtained. For Q = 200 µL/min, the shape remains mostly circular, but some effects due to reaction and diffusion deformed the initially symmetrical profile. For Q = 5 µL/min, patterns start to be observed forming ramifications around the initially circular shape as described in Fig. 4. For Q = 2.5 µL/min, a fractal-like structure is observed in the displacing solution. Diffusive effects are remarkable for the lower flow rate case. This is observed in the loss of coloration in the displacing solution. The yellowish reaction zone is also observed for all cases, being enlarged for the lower flow rate cases.

Nevertheless, when a change in pH is chemically induced at the interface, the phenomenon is quite different as observed in Fig. 4 (upper row labeled Reactive). Again, the more viscous solution (A) is being injected through the centrally located orifice in the Hele–Shaw cell. Note that almost instantly, the circular geometry of the interface is broken, and some fingers are observed. These fingers grow non-symmetrically until one of them reaches the outer boundary of the cell and the experiment ends. We note a strong dependence of this instability on the inflow rate. As the flow rate of solution A is increased, the instability disappears and a regular circular interface is displayed again. We studied this dependence in Fig. 5 where exactly the same experiment is repeated at different flow rates (only the final configuration of the interface is shown for each case). Larger flow rates produce circular interfaces while for low values of the flow rate the instability appears due to the chemical reaction at the interface.

We conclude that this instability is not a typical fingering instability that it is produced due to the viscous difference at the interface and becomes more unstable as the displacement velocity is increased^10,26^. In our case, the interfacial reaction is crucial to trigger the instability. Apart from the non-reactive control experiment presented in Fig. 4 that shows that the absence of gluconic acid in solution B prevents the instability, several other control experiments were performed and shown in the SI. There, the different reactants were replaced by water and in most cases, part of or the entire interfacial reaction is stopped inhibiting the formation of patterns. Only when the pH color indicator was replaced by water, the instability was observed unaltered (the observation, then, was done using Schlieren techniques described in the Methods section). The conclusion from all these experiments is that the interfacial instability is triggered and maintained by the reactivity at the interface between the two solutions.

**Figure 6:**
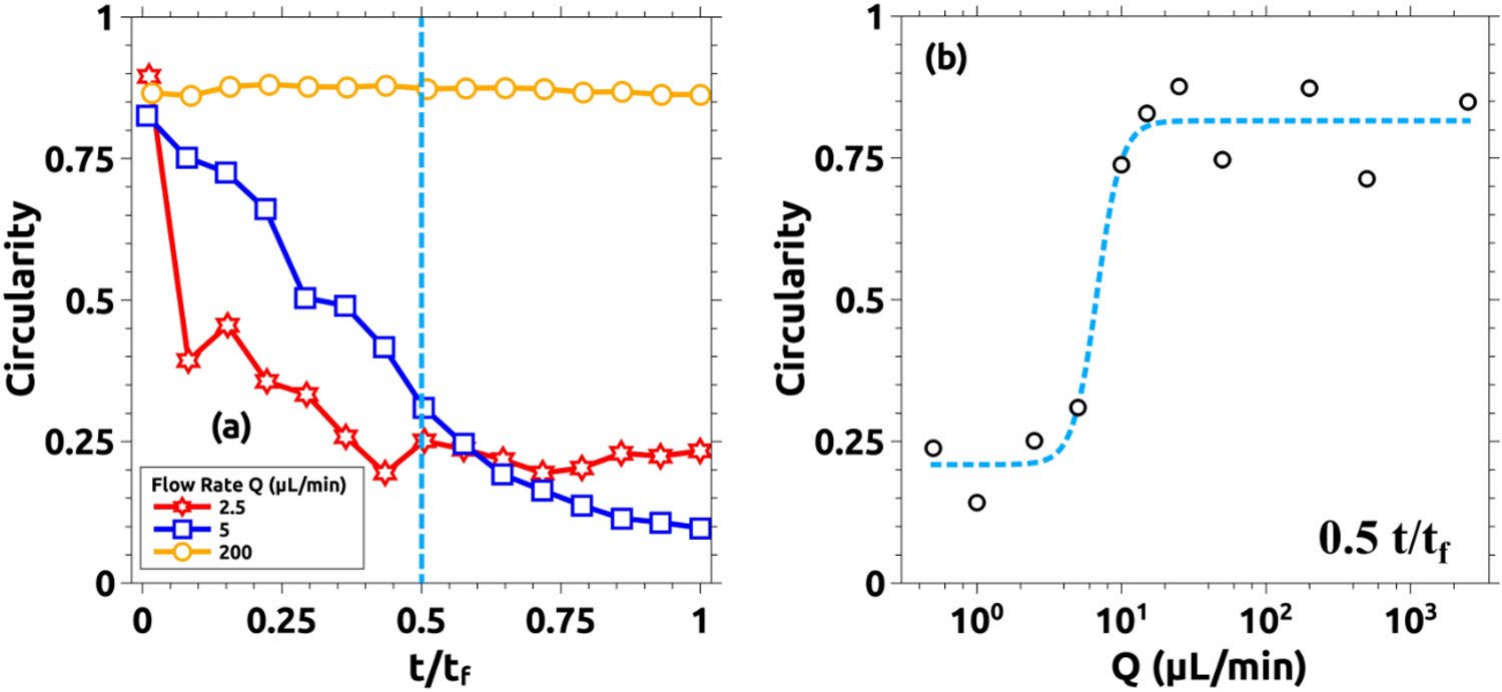
Quantitative comparison of the displacing profile as a function of the flow rate. The shape of the displacing front is described by the circularity of the pattern as described in the methods section. (**a**) Variation of the circularity versus the normalized time for Q = 200, 5, and 2.5 µL/min. For the larger flow rate, the circularity is closer to 1, meaning that the shape of the front remains close to a perfect circle. As Q decreases, the initially stable displacing front (with circularity values close to 1 when t/t_f_ $\cong$ 0), changes its morphology, and the circularity drops. Those changes become more pronounced for lower flow rates (Q = 2.5 µL/min in the figure). Here, circularity decreases till t/t_f_ $\approx$ 0.5 that reaches a stationary value around 0.25 once the pattern is fully developed. (**b**) Semi-log plot of the circularity of all the cases studied at t/t_f_ = 0.5 (light-blue dashed line indicated in (**a**)). For larger values of Q, the circularity remains closer to 1 until 10 µL/min. For lower flow rates, the circularity value drops down to 0.25. Only three cases are presented in (**a**) for clarification. The remaining reactive cases in addition to the control experiments are also analyzed and are presented as supporting information.

A multitude of experiments was performed to characterize this result and they are summarized in Fig. 6. Figure 6a presents the variation of the interface circularity as a function of time for different flow rates. The circularity (calculated as described in the Methods section) provides a value of how close the interface is to a perfect circle (characterized by a circularity value of 1). This value is plotted versus time normalized by the total time of each experiment (time at which the solution A reaches the outer boundary of the Hele–Shaw cell). Note that for low values of the flow rate, the circularity immediately drops to low values. When the flow rate is closer to the transition but still in the instability region, the circularity drops as well but it needs more time to drop. This seems reasonable because as time goes, the average radius of the interface becomes larger and so the linear velocity of the interface becomes smaller. Thus, we move deeper into the instability region. Larger values of the flow rate and clearly out of the instability region are characterized by a constant value of the circularity very close to 1. Figure 6b plots the values of the circularity in the middle of each experiment versus the flow rate. Note that values of the flow rate above Q = 10 mL/min fail to induce instability in the system. Different properties can be measured at the interface to characterize it^4,22,27^. We choose the circularity because it straightforwardly presents the results^8^. A brief description of other properties and some calculations are in the SI.

### Mechanism of the induced instability

**Figure 7:**
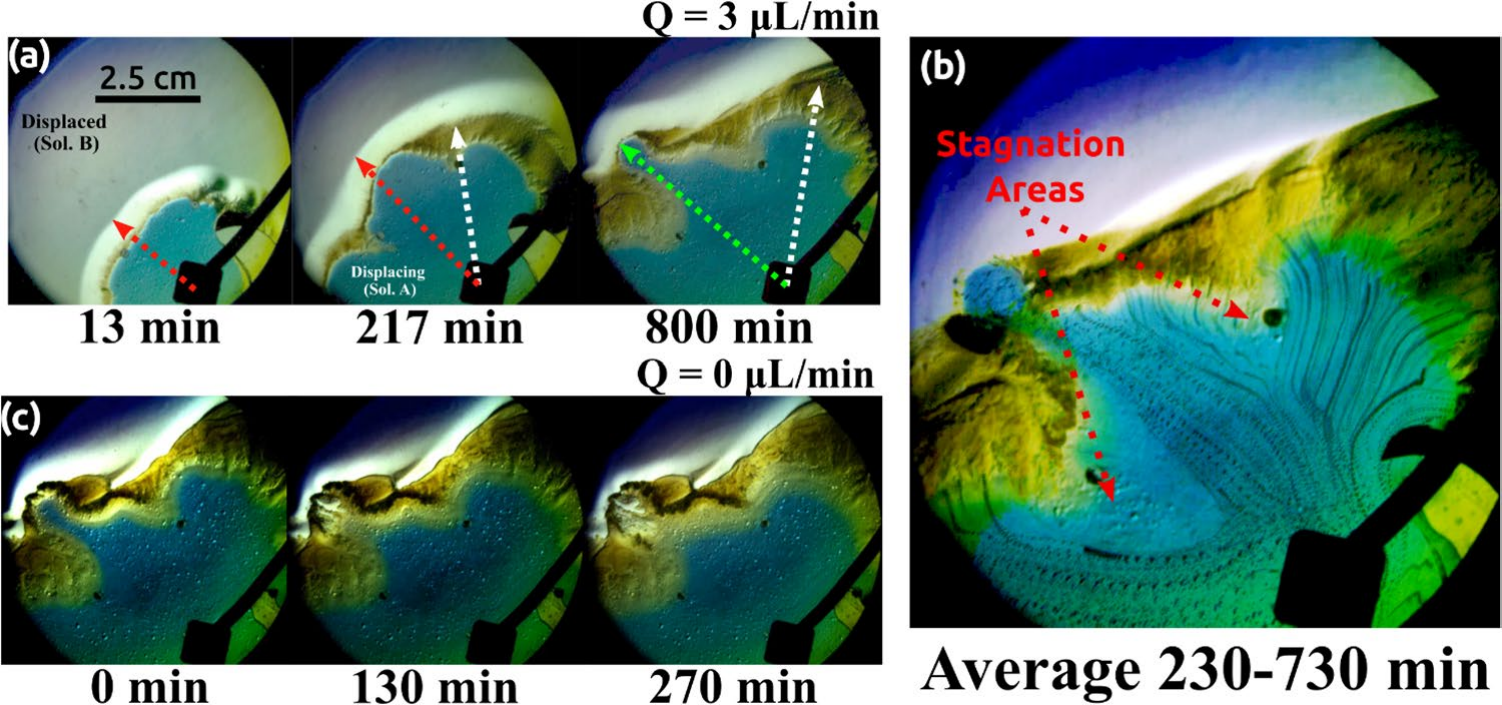
(**a**) Schlieren visualization for an experiment with Q = 3 µL/min. Red dashed arrows indicate the diffusive interfacial zone while the white dashed lines indicate the brownish crust generated by the contact of solutions A and B. The green dashed line indicates one of the fingers observed in Fig. 4. (**b**) Average flow path of the displacing solution obtained by averaging the experimental frames in the range of time between 230 and 730 min via image processing techniques. The fingers are generated by a synergy between different processes. In the first place, there is an inhomogeneous distribution of the displacing solution driven by a low flow rate and the accumulation of the brownish crust. This flow inhomogeneity facilitates the generation of stagnation areas as indicated in the figure. The crust accumulation produces a stopper that locally reduces the permeability and changes the pressure field preventing the natural flow of the displacing solution. Once the pressure is above a certain value, solution A is ejected through one of the flow paths. Once the crust is penetrated, displacing and displaced solutions start to react producing more crust. (**c**) A reaction front is observed when the injection is stopped. This front moves opposite to the flow direction and it is responsible for the finger to move backward.

We use the Schlieren technique^8,16–18,28^ (described in the Methods section) to track changes in the optical index of refraction of the solutions induced by fluid movements that cannot be observed by a direct optical inspection. The use of this technique makes possible the observation of phenomena that are difficult to see with a direct setup. Figure 7 shows pictures of an experiment with a flow rate Q = 3 µL/min. Once the displacing solution is injected (Fig. 7a), both solutions start to react, creating a brownish crust at the interface between both fluids. This crust locally reduces the permeability and increases the pressure inside the cell. This crust produces stagnation areas where more precipitation accumulates. As the displacement solution continues to be injected into de cell, the pressure is increased and, eventually, due to some symmetry breaking mechanism, it is released through some cracks in the crust. This process causes the characteristic patterns observed in the previous figures. The displacing solution contacts, then, fresh displaced solution, and the interfacial reaction starts again, thus, producing more new crust that eventually stops the propagation till the whole process is repeated. This is better appreciated in Fig. 7b in which all the experimental frames between 230 and 730 min were averaged into one single image by image projection methods^15^. This provides dynamic information about the displacing flow in one static image and shows the symmetry-breaking process produced by the accumulation of crust in those regions in which the displacing does not flow. The crust formation is produced by the agglomeration and subsequent precipitation of PAA molecules driven by the acidic pH of the gluconic acid^29^. In this relatively high acidic condition (pH ≈ 2), the PAA molecules become fully protonated favoring intra- and intermolecular hydrogen bond formation. This phenomenon produces the PAA molecules to aggregate and precipitate almost instantaneously inside the Hele–Shaw cell at the interface. This process was previously reported in Escala *et al*^8,14^ in a system with a similar composition. More information regarding the precipitation formation is included in SI.

Figure 7c shows another mechanism that is observed to play a major role. Once the injection is stopped a chemical reaction wave is observed to propagate in the direction opposite to the previous flow velocity. In fact, this chemical wavefront is always present in these experiments and it is responsible for the dynamics of the fingers.

The mechanism of the chemical front needs two conditions to occur. One condition is that SO_3_^2−^ must be in solution A (see Supp. Info. for more details). The acid pH of the gluconic acid solution interacts with the SO_3_^2−^ of solution A and producing HSO_3_^**−**^ (which is essentially acid) by equilibrium displacement as expressed by the following equations^8,14^:

3a $$PAA \leftrightarrow PA^{ - } + H^{ + }$$

3b $$SO_{3}^{2 - } + H^{ + } \leftrightarrow HSO_{3}^{ - }$$

where $PA^{ - }$ is the polycarboxylate ion.

It is also important to note that there is a large gradient of protons between solutions A (pH ≈ 12) and B (pH ≈ 2). This explains why the observed chemical front is indeed an acid front, and the color indicator switches from blue (basic state) to yellow (acid state) (see Figs. 5 and S16 in Supp. Info). The second condition for the reaction front to occur is the existence of a large difference in diffusivity between the polymer molecules (D_PAA_ = 1 × 10^–11^ m^2^/s)^4,8,21,23^ and the H^+^ (D_H_^+^  = 9.3 × 10^–9^ m^2^/s)^24,25^ of the solution B. This difference is enhanced by the effect in the proton apparent diffusivity produced by the PAA molecules which act as a reversible proton acceptor^30,31^. This difference in diffusivity plays a major role as a condition for the front propagation when autocatalysis is not possible. As no oxidant species are in the medium, the nature of this chemical front is far from the classical autocatalytic approach vastly observed in many pH-oscillators^30,31^. Diffusion also plays an important role in the system stability^32^ (See SI for more details).

**Figure 8:**
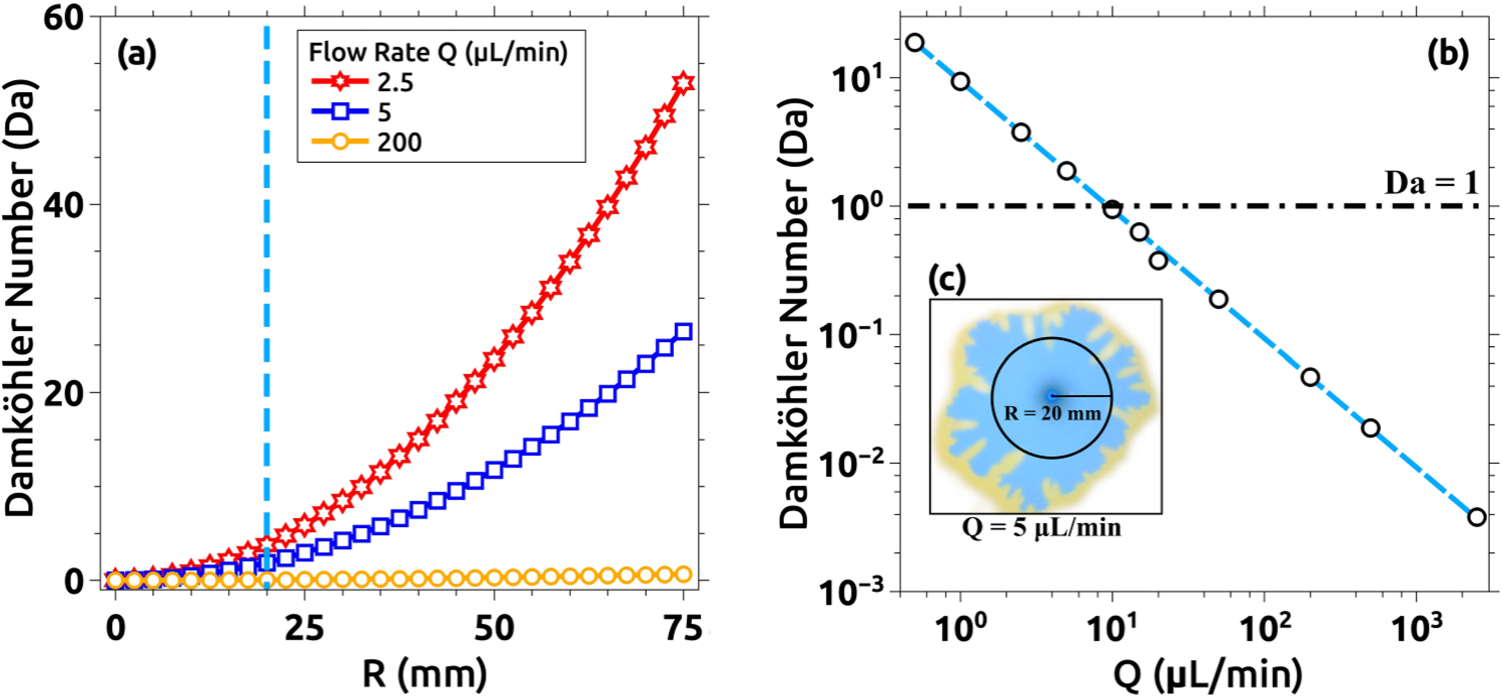
Damhköler number (Da) estimation as a function of the average interface radius (R). (**a**) Comparison of Da for Q = 2.5, 5, and 200 µL/min versus the average interface radius. A remarkable increment in the Damhköler numbers is observed for lower flow rates indicating that the reaction process is faster compared to the advective process. Da increases proportionally to the radius and inversely proportional to the flow rate. Both observations are logical as the velocity of the displacing solution decreases with the radius in a radial Hele–Shaw cell. This effect is more prominent if the flow rate is decreased allowing reaction and advection to have comparable timescales for a certain R. Only 3 cases are presented to facilitate visualization. (**b**) Log–log plot of Da as a function of Q for all the cases studied. All values are taken at R = 20 mm, which is the average radius of the circumference delimited by the region before the blue-yellow boundary as exemplified in (**c**) for Q = 5 µL/min. Results are coherent with the experimental analysis presented in Fig. 6b. For Q > 5 µL/min, Da < 1 indicating the dominance of the advective process, while for Q < 5 µL/min, Da > 1 indicating the predominance of the reactive process.

From these observations, it seems straightforward that there must be a coupling between the flow velocity and the chemical rate. In order to stress this, we estimated the non-dimensional Damköhler number following^8,22,27^ (see Supp. Info for more details). Its physical meaning is just the ratio of the hydrodynamic characteristic time over the temporal scale for the reactive processes involved. Figure 8a plots the estimation of the Damhköler number (Da) as a function of the average radius of the interface for three characteristic flow rates. The average interface radius (R) (Fig. 8c) is defined as the minimum radius at the blue-yellow boundary in the first experiment in which pattern formation was observed (Q = 5 μL/min). Note that as the experiment evolves, the interface average radius becomes larger and so the hydrodynamic temporal scale becomes smaller and Da larger. Figure 8b plots all the Damhköler numbers estimated for all the experiments performed calculated for an average interface radius of 20 mm. For Q > 10 µL/min, Da < 1 indicating the prevalence of the advective process, while for Q < 10 µL/min, Da > 1 demonstrating the prevalence of the reactive process. Da >  > 1 for Q = 0.5 µL/min and Da <  < 1 for Q = 2500 µL/min are the extreme cases that confirm the tendencies. We conclude that for larger injection flow rates, the hydrodynamics is such that the reaction front has no practical effect and the normal behavior is observed (no instability). Only when the reaction is allowed to play a role (Da > 1) the instability is seen.

These results demonstrate the physical aspects of pattern generation. They also show that there are two chemical processes involved playing a major role in pattern formation and directly affecting the physics of the system. Once solution A is injected, there is a reaction front that moves opposite to the flow direction. As this is a radial system, the flow velocity decreases with the radius. Once the injection velocity matches the velocity of the chemical front, the crust starts to accumulate and, thus, reduces locally the permeability of the system. This permeability loss locally increases the pressure making the displacing fluid break the crust and be ejected through the cracks generating the characteristic fingers. When both fluids make contact again, the reaction front forces the finger to recoil. The whole process is better appreciated in the movies included as supplemental info.

### Reverse experiment: less viscous solution displaces a viscous solution

**Figure 9:**
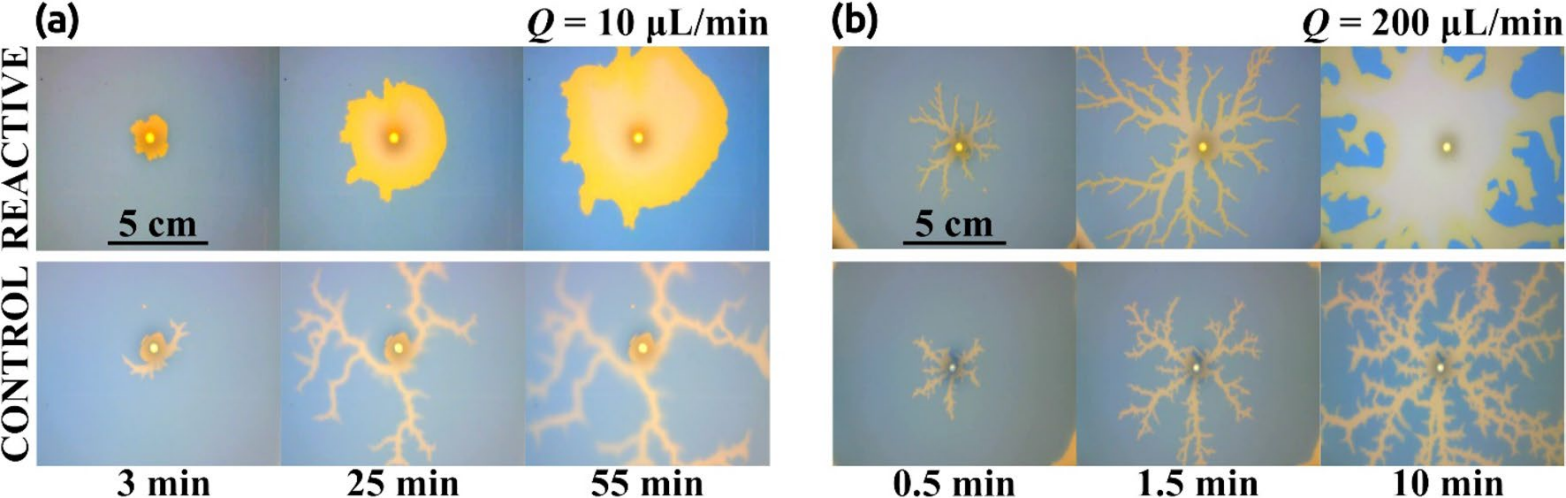
Stabilization of an initially unstable front for (**a**) Q = 10 mL/min and (**b**) Q = 200 mL/min. In both flow cases, a reactive (upper row) and a non-reactive (lower row) experiment were conducted. Non-reactive cases (control) are always unstable leading to the development of a fractal pattern that rapidly reaches the border of the observation region. The reactive cases are sensitive to the flow rate Q, in (**a**) the displacing front growths stable showing a more circular pattern. When the flow rate is increased (**b**), a fractal shape is observed again. Note that in this case, once the border of the reactor is reached, the fractal ramifications increase their thickness changing the front shape. This is not observed in the control case, where once the pattern reaches the border of the reactor, it remains fractal.

We consider in this section the reverse situation. The more viscous solution (solution A) is located initially inside the Hele–Shaw cell and the less viscous solution (solution B) is injected into the cell with a constant flow rate becoming, thus, the displacing solution. This is an unstable situation from a fluidic point of view and in the absence of reaction produces a viscous fingering instability that has been widely characterized in the literature and appears in many industrial applications with negative impact^7,33,34^. We analyze here the role played by the interfacial reaction in controlling the instability. Figure 9 shows the results of such an experiment for two different values of the injection velocity. Figure 9a (lower row) presents the non-reactive control experiment where the injected solution is replaced by distilled water. This is an unstable configuration and viscous fingering is immediately observed. The upper row presents the case where the interfacial reaction is allowed (the displacing solution is gluconic acid as described in the Methods section). In this case, the evolution is completely different, the instability is suppressed and there is an effective displacement of the more viscous solution A. In Fig. 9b, the same experiments are repeated but at larger flow rates. Note that in this case, the instability is not suppressed by the interfacial reaction and it is observed both in the control experiment and in the reactive one.

**Figure 10:**
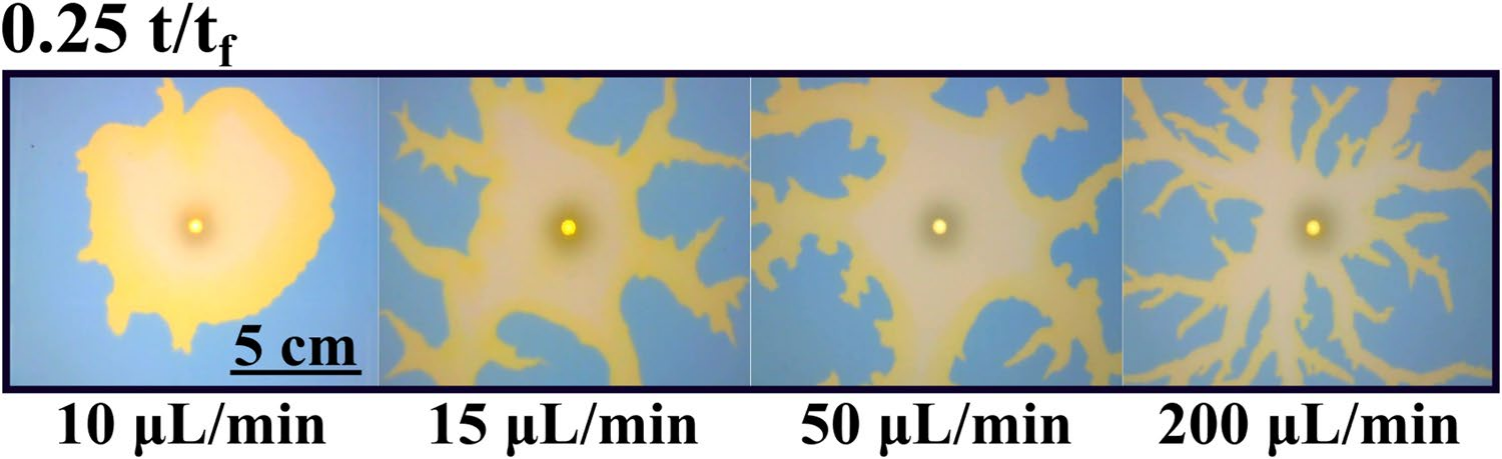
Reacting interfaces for several flow rates (Q). All images are taken at t/t_f_ = 0.25 for each case. As the flow rate is decreased, the initially unstable front changes from a fractal-like structure into a more stable circular shape. For intermediate flows (Q = 15 and 50 µL/min) the fractal geometry is still there but the fractal fingers get enlarged due to the reactive process. Only for the lower flow rate case (Q = 10 µL/min), the interface becomes rounded and stable.

**Figure 11:**
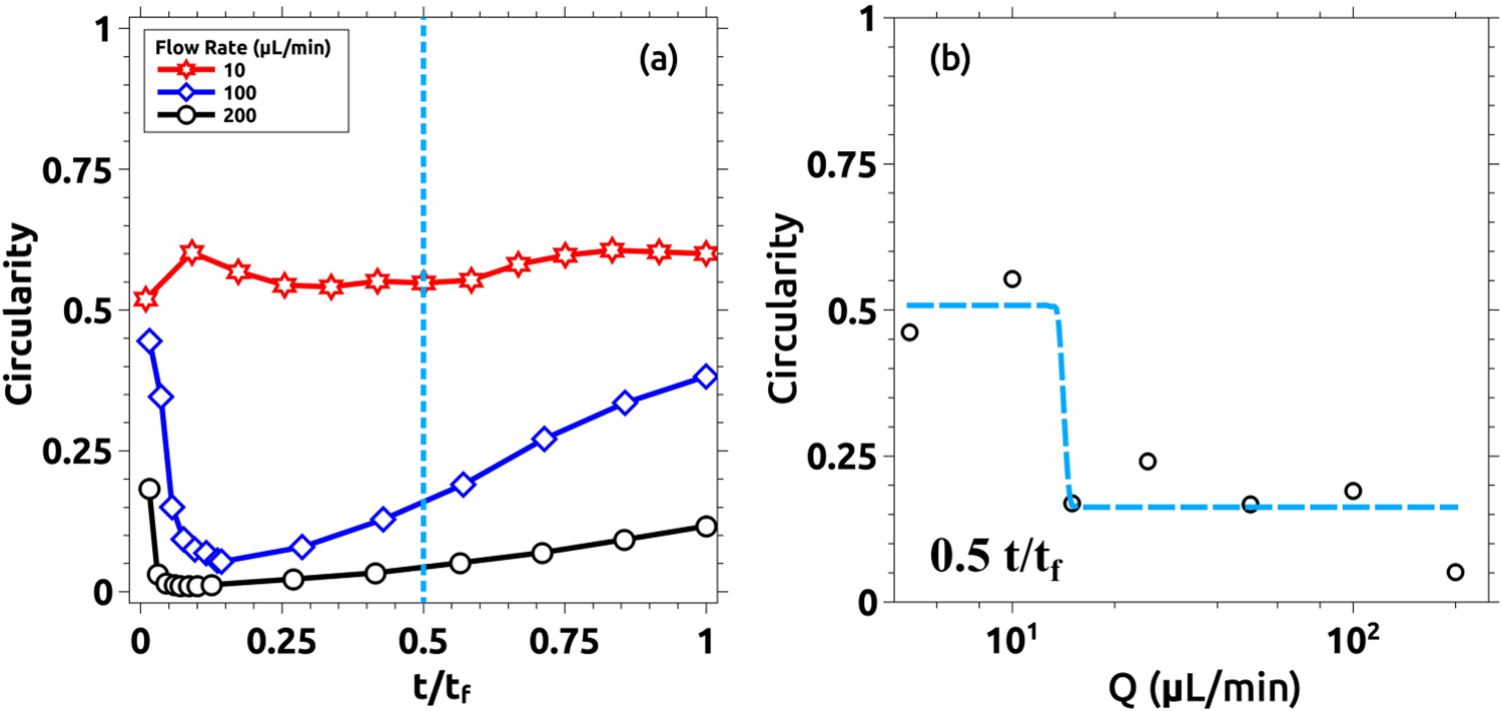
Quantitative measurements of the morphological changes of the interface as a function of the flow rate Q. (**a**) Evolution of the circularity for three different flow rates: 10, 100, and 200 µL/min. For Q = 100 and 200 µL/min, the circularity rapidly drops to values near zero for t/t_f_ < 0.25. This is due to the fractality of the displacing front. For t/t_f_ > 0.25, the circularity increases with time due to reactive effects as shown in Fig. 9b. For Q = 10 µL/min, the circularity remains stable between 0.5 and 0.6 during the recorded time. Only some selected cases are plotted here for clarity (see the rest, together with the corresponding control experiments, at the SI). (**b**) Semi-log plot of the circularity for all the studied cases at t/t_f_ = 0.5. The system becomes more unstable as the flow rate increases. For Q = 10 and 5 µL/min, the system becomes more stable due to the interfacial reaction.

Similar to Fig. 5, the effect of the flow rate is observed in Fig. 10. Here, snapshots taken for different experiments with different flow rates are compared. Experiments were run (as explained in the Methods section) till the displacing solution touches the external wall (labeled t_f_ in the manuscript and figures). In this figure, we compare pictures taken at 0.25 t_f_. Note that for Q > 10 mL/min the interface becomes unstable. This corresponds with Damköhler numbers below 1 as described in the previous section. We characterize the intensity of the instability by measuring the circularity of the interface and the results are plotted in Fig. 11. Figure 11a presents the variation of the circularity along with the experiment for three different flow rates. For Q = 10 mL/min (red line in Fig. 11a) the value of circularity is high and almost constant during the entire experiment. In the two other cases, the interfacial reactions are not able to suppress the instability, and circularity drops to very low values (close to zero). As time evolves, circularity increases due to a combination of diffusive effects and the chemical front previously described. Note that in this case, the reaction front moves in the same direction as the flow rate. Figure 11b plots the values of the circularity for several flow rates measured at t = 0.5 t_f_. There is a clear transition as the flow rate is increased (above 10 mL/min), below this critical flow rate the fingering instability is suppressed while above the instability is still present. Note that this transition is marked by the Damköhler number. Da > 1 corresponds to Q < 10 mL/min and a stable interface is observed while for Da < 1 (corresponding to Q > 10 mL/min) the interface is unstable.

**Figure 12:**
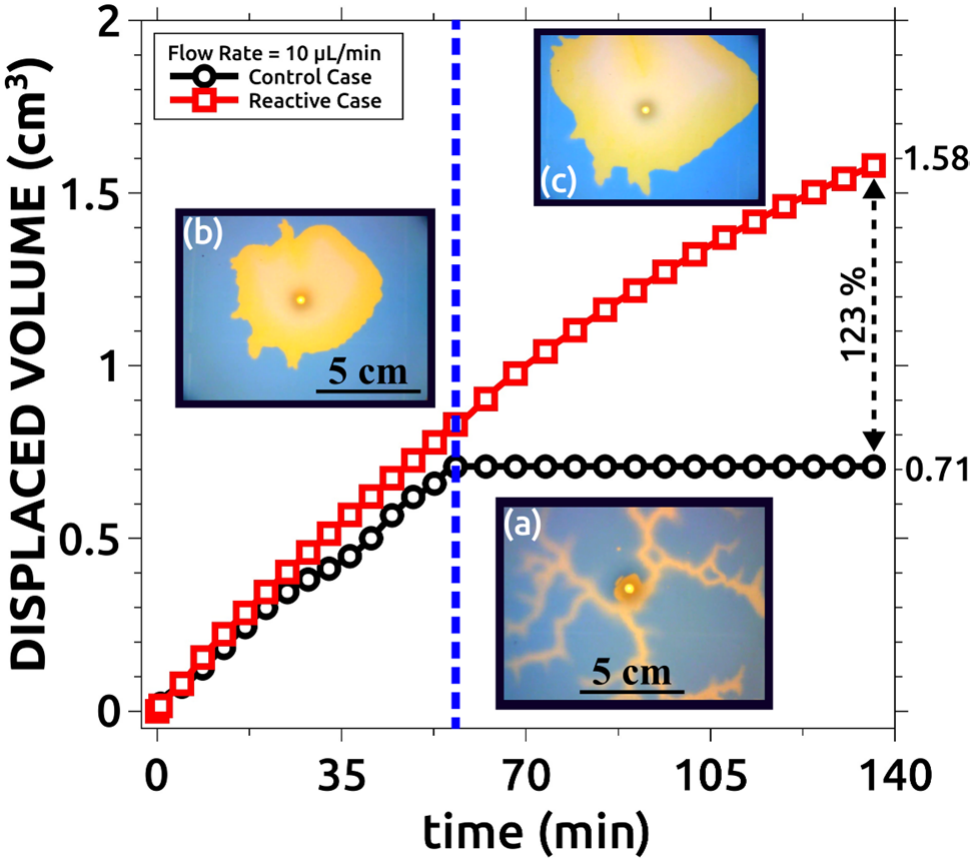
Comparison between the displaced volumes for the reactive and control cases for Q = 10 µL/min. The reactive case displaces more than twice the fluid displaced by the non-reactive case. The center vertical line indicates the time when the pattern in the control experiment reaches the reactor boundary (**a**). Snapshot (**b**) was taken at the same time as (**a**) but for the reactive case. Snapshot (**c**) was taken at the end of the reactive case experiment.

Another important feature, especially relevant for industrial applications, is the total amount of displaced solution per experiment. This is shown in Fig. 12 for the control case without reaction and the reactive situation (Q = 10 mL/min in both cases). Note that once the control case, with fingering instability, reaches the cell boundary, solution A stops being displaced (black curve in the figure). On the other hand, when the reactive situation is considered and the instability is suppressed, the displacement of solution A continues for larger times, and the total volume of solution A displaced is significantly larger (an additional 123% more in our case. Red curve in the figure).

**Figure 13:**
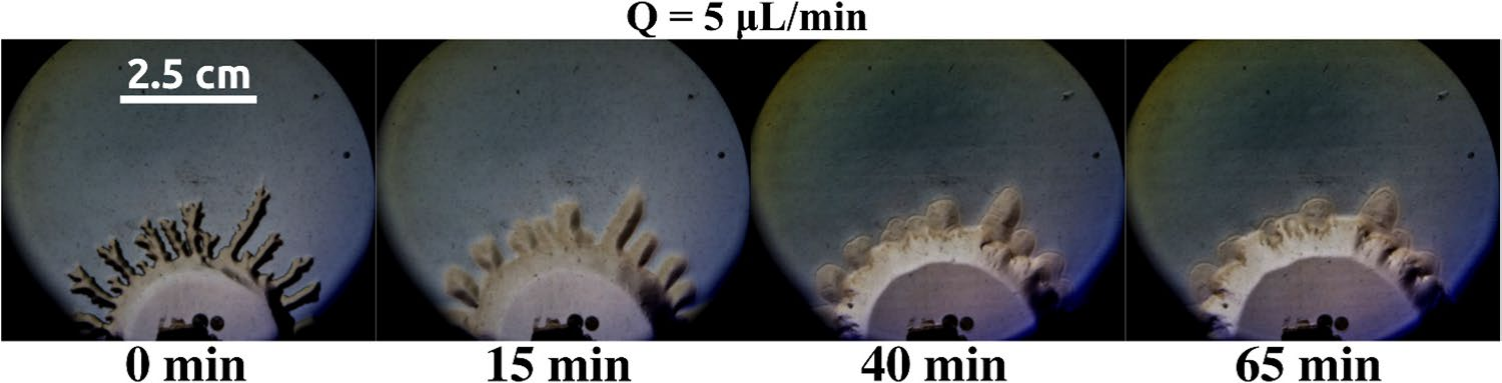
Front stabilization mechanism observed using the Schlieren technique. Starting from an initially unstable situation, the stability of the system is increased by the synergy between the polymer aggregation (reactive front) and the flow displacement. This is only observable when the reactive and flow fronts move synergistically.

Similar to the previous case, we also use the Schlieren view to understand the stabilization mechanism. The stabilization process of an initially unstable configuration is shown in Fig. 13 through the Schlieren technique. Note that the mechanism proposed for the direct experiment above is still valid in the present case. For low flow rates, where chemical and advective timescales are comparable (Da≈1), the chemical front moves synergistically with the flow front. The reaction produces polymer precipitation and the creation of an effective crust. As pressure increases due to the incoming flow, the crust breaks. At this point, the faster reaction rebuilds the crust. This process, repeated randomly around the interface, results in a stable rounded interface propagating smoothly. When the flow rate is increased (and the Damköhler number is smaller than 1), the fluid moves faster than the reaction characteristic time, and no effective wall is created, thus, the system remains unstable (see S.I. for some pictures).

### Mathematical model and numerical solutions

Here, we incorporate the mechanism described above into a mathematical model that couples the dynamics of the displacement of reactive fluids in a porous media inside a Hele–Shaw cell, the existence of a chemical front, and polymer precipitation that locally affects the permeability of the system. We considered parameter values as measured experimentally.

For a system with viscosity µ, porosity ϕ and permeability κ, the governing equations, based on Darcy’s Law for incompressible fluids, are given by the following Reaction–Diffusion-Advection set of equations^10^,

4a $$\nabla \cdot \vec{u} = 0$$

4b $$\nabla p = - \frac{{{\upmu }\left( {{\text{A}},{\text{B}}} \right)}}{\kappa \left( C \right)}\vec{u}$$

4c $$\varphi \frac{\partial A}{{\partial t}} + \vec{u} \cdot \nabla A = \varphi D_{A} \nabla^{2} A - k_{1} A B - k_{2} A B$$

4d $$\varphi \frac{\partial B}{{\partial t}} + \vec{u} \cdot \nabla B = \varphi D_{B} \nabla^{2} B - k_{2} A B$$

4e $$\varphi \frac{\partial C}{{\partial t}} + \vec{u} \cdot \nabla C = \varphi D_{C} \nabla^{2} C + k_{2} A B$$

where $\vec{r} = \left( {x,y} \right)$ is the position vector. We considered a simplified version of the reactive solutions where A stands for solution A containing the polymeric solution, B for the solution B (containing the gluconic acid), and C for the polymer precipitate. Note that species A, B, and C depend both on space location and time, $A = A\left( {\vec{r},t} \right)$, $B = B\left( {\vec{r},t} \right)$ and $C = C\left( {\vec{r},t} \right)$. ϕ is the porosity of the system and it is set constant for a Hele–Shaw cell^8,10,26,32^. D_A_, D_B_, and D_C_ are the diffusion coefficients of A, B, and C respectively. We assume that the polymeric solution (A) diffuses slower in comparison with the acidic solution (B) and that the precipitate (C) does not diffuse at all^8,23,35,36^.

The permeability of the system κ(C) depends on the polymer precipitate and it is modeled by the following equation^35^,

5 $$\kappa \left( C \right) = {\upkappa }_{0} e^{{ - R_{\kappa } \left( {C/C_{m} } \right)}}$$

where κ_0_ is the permeability when C = 0 (no precipitate in the medium), and it is calculated as κ_0_ = *h*^2^/12^26,37–39^, and *h* is the separation gap of the Hele–Shaw cell. We define κ_m_ = κ(C_m_) as the permeability when C = C_m_ and *R*_*κ*_ = ln(M_0_/M_m_) as the permeability log-mobility ratio, where M_0_ = κ_0_/μ and M_m_ = κ_m_/μ are the mobilities when C = 0 and C = C_m_, respectively. C_m_ is a reference value that we choose as the maximum possible concentration of the species C in the medium. The parameter *R*_*κ*_ quantifies the influence of precipitation on permeability changes. A positive value of *R*_*κ*_ indicates that C reduces the permeability of the porous matrix locally^35^. This is the situation assumed in our case, as the precipitate formation clogs the cell reducing the permeability. For this work, a value of *R*_*k*_ = 80 is chosen ad-hoc based on experimental observations.

We consider that the viscosity of the system µ(A, B) is only affected by the displacing and displaced solutions and it is defined by the following relation^10^:

6 $$\mu \left( {A,B} \right) = \mu_{A} e^{{R_{B} B/A_{0} }}$$

where μ_A_ = μ(A_0_,0,0), represents the viscosity of the polymeric solution for a specific concentration A_0_ in the absence of any other solution in the medium. The parameter *R*_*B*_ compares the viscosity of the two reactant solutions^10^. Following the experiments, both parameters *µ*_*A*_ and *R*_*B*_ are fixed, and the stability of the system is only affected by the initial configuration. Those values are also estimated from experimental observations.

A simplified model is used to simulate the reactivity between the displacing and displaced solutions. This model is based on the mechanisms proposed in the previous section and it is given by the following set of chemical equations:

7 $$A + B \mathop \to \limits^{{k_{1} }} B + D$$

8 $$A + B \mathop \to \limits^{{k_{2} }} C$$

where Eq. (7) models the reactive front. It represents the bisulfite generation due to the acidity of solution B in one single step. D represents any secondary product species generated by this mechanism and it is included to preserve mass conservation. By sake of simplicity, the concentration of D is set constant and equal to zero during the entire simulation.

Equation (8) models the polymer precipitation (C) that is produced when both solutions (A and B) make contact at the interface. As previously mentioned, the polymer precipitation is produced by aggregation of PAA molecules due to a low pH interfacial condition^29^. The use of such an equation to model precipitation has been extensively used in many previous works^10,28,35,40,41^. *k*_*1*_ and *k*_*2*_ are the rate constants. *k*_*1*_ was calculated from the experimental front velocity (see Supp. Info for more details). The value of *k*_*2*_ was set as *k*_*2*_ = *k*_*1*_/10 to better fit the experimental observations.

**Figure 14:**
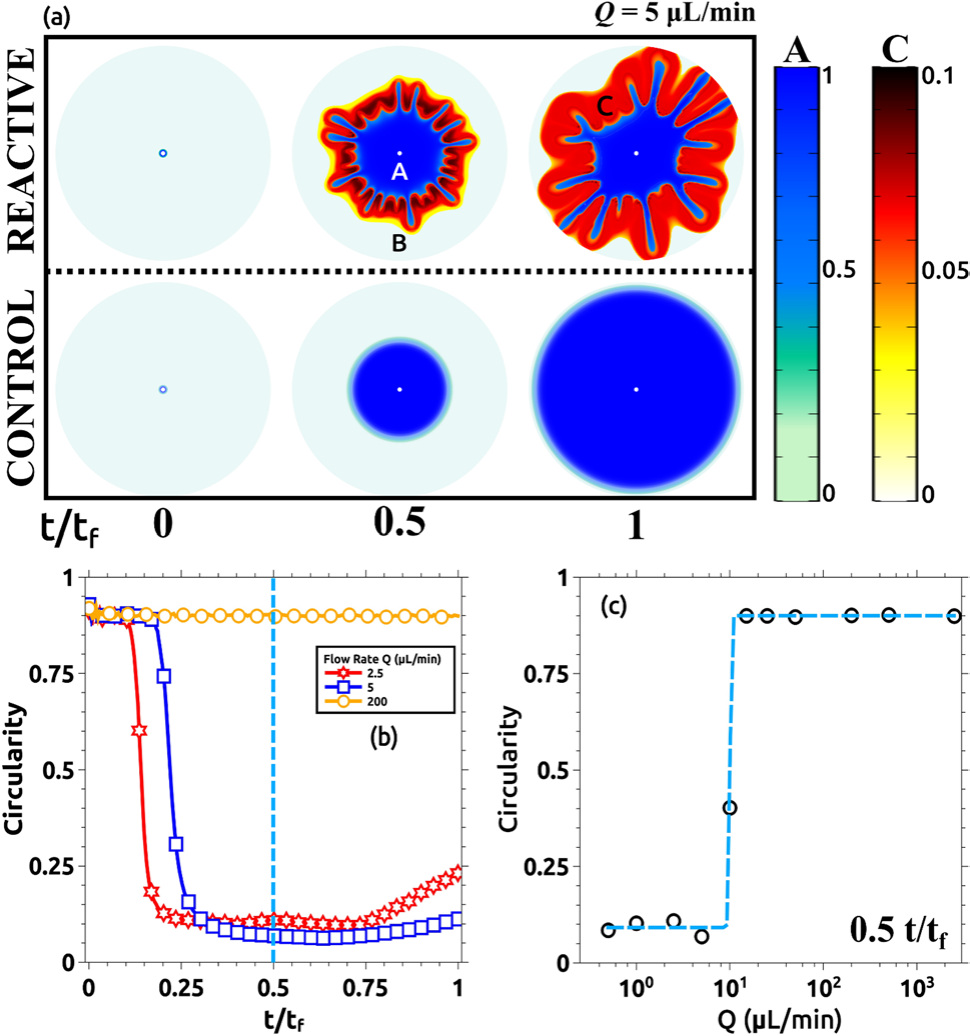
Numerical results of the simulation with the direct experiment configuration. (**a**) Numerical representation of the concentration fields of solutions A, B, and the precipitate C in a similar way as in the experimental Fig. 4. The concentrations of A and C are indicated by the corresponding color bars. The upper row shows the reactive case and the lower row shows the control case where solutions A and B do not react. Color codes are as follows: blue shows maximum of A concentration while light green is for the whole domain occupied by B specie, the concentration of the precipitate (overlayered in the same figure) goes from white (no precipitate) to red (maximum of precipitate). All results are presented in normalized time. (**b**) Circularity variation of the same three representative cases presented in the experimental Fig. 6a as a function of the normalized time (t/t_f_). The remaining cases are shown in the SI. (**c**) Circularity variation of all simulated cases as a function of the flow rate Q at t/t_f_ = 0.5. Simulation parameters are indicated in Table 1.

This set of equations is integrated as described in the Methods Section. Figure 14 presents the numerical results obtained for the direct experiment. Figure 14a shows the overlaid image of the concentration fields of species A and C for Q = 5 µL/min. Note that both the pattern structure and the polymer precipitation are qualitatively well reproduced by the model (in the S.I. some animations of this simulation are shown with a good agreement with the experimental ones). In the non-reactive case (lower row in Fig. 14a, labeled as control), the system remained stable as in the experimental counterpart. Figure 14b shows the circularity evolution in normalized time (t/t_f_) for three representative cases (the remaining cases are presented as supplementary information) in good agreement with the experimental results in Fig. 6. When the flow rate is increased, the system reactivity results diminished compared to the advection and the system remains stable (circularity ≈ 1). For lower flow rates, the chemical timescales match the advective characteristic times at some specific radius. This, in combination with the local reduction of permeability (and the subsequent pressure increment) produced by the precipitation, leads to the fingering formation. Note that in the simulations the initial circularity is always close to 1.

The circularity variation as a function of the flow rate is presented in Fig. 14c, also in good agreement with experiments.

**Figure 15:**
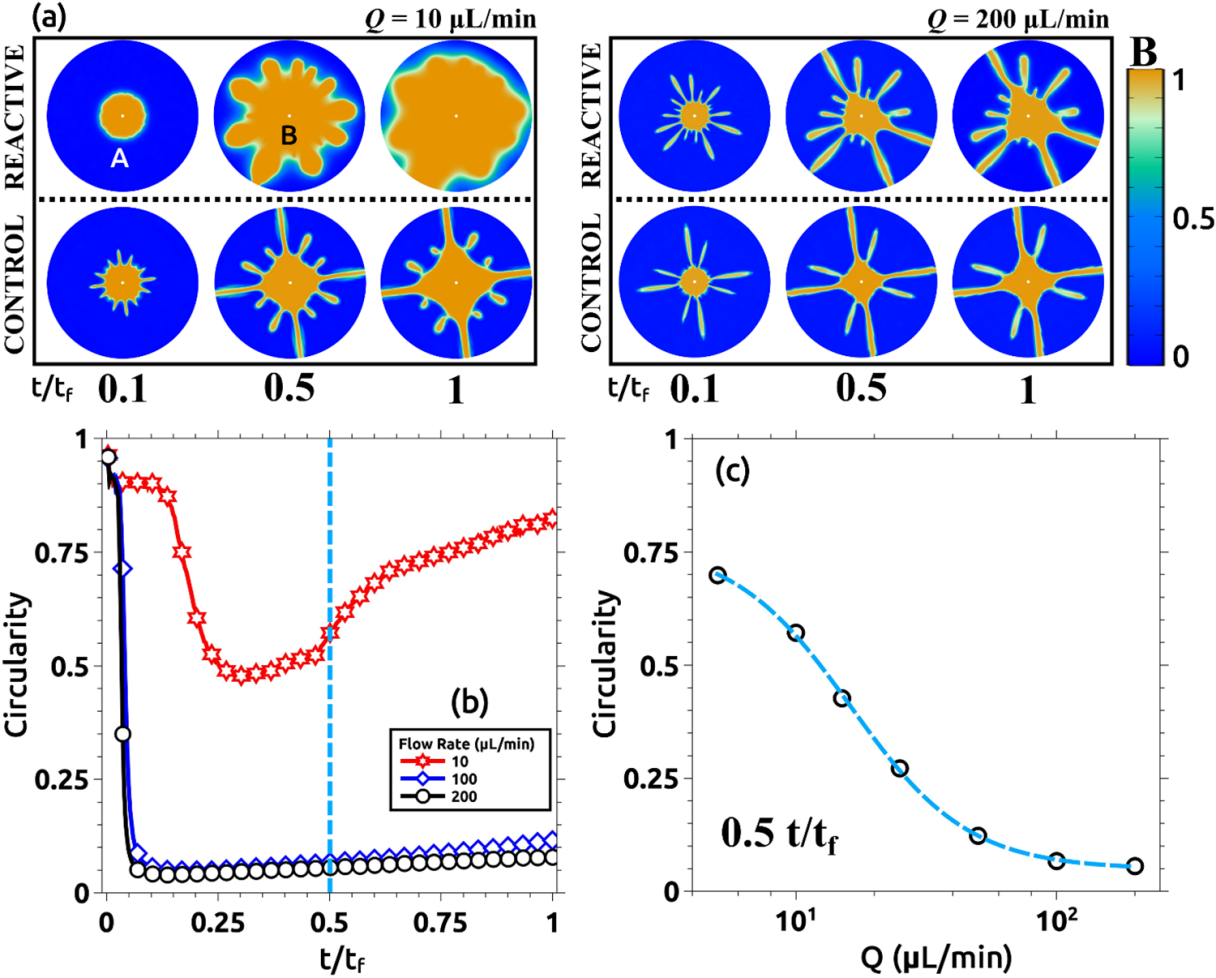
Numerical results of the simulation of the inverse experiment. (**a**) Numerical representation of the concentration fields of solutions A and B similar to the experimental Fig. 9. The upper row shows the reactive case for two different flow rates Q = 10 and 200 ul/min. The lower row shows the control case where solutions A and B do not react for the same flow rates. The color bar indicates the minimum (blue) and maximum (yellow) of B concentration. All results are presented in normalized time. (**b**) Circularity variation of the same three representative cases presented in the experimental Fig. 11a as a function of the normalized time (t/t_f_). (**c**) Circularity variation of all simulated cases as a function of the flow rate Q at t/t_f_ = 0.5. The simulation parameters are indicated in Table 1.

Figure 15 shows the numerical results for the reverse case. Figure 15a shows a comparison between two different flow rates Q = 10 and 200 µL/min for both reactive (upper row) and non-reactive (lower row) cases in normalized time. Figure 15b shows the circularity variation versus the normalized time for three representative flow rates (the remaining cases are included as SI). Figure 15c shows the circularity variation as a function of the flow rate at t/t_f_ = 0.5. Note that the results are qualitatively and almost quantitatively equivalent to those observed experimentally in Figs. 9 and 10.

**Figure 16:**
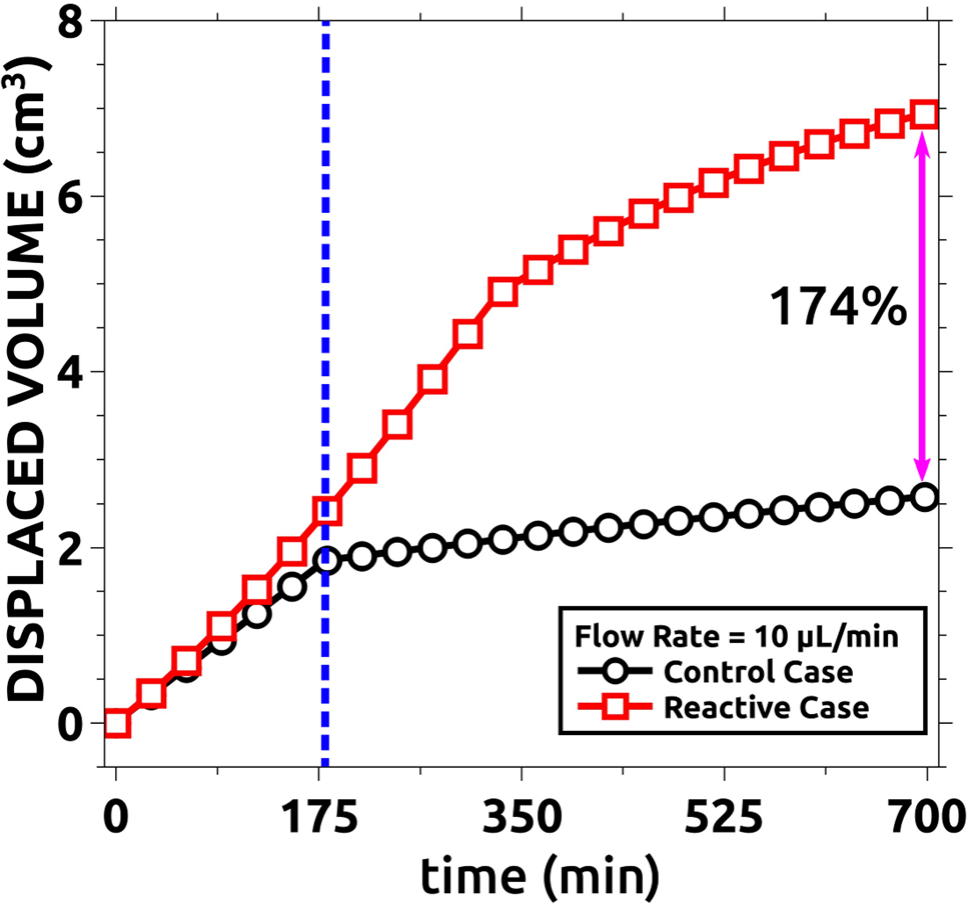
Quantitative numerical comparison between the displaced volumes for the reactive and control cases when Q = 10 µL/min. The total displaced volume is compared at the end of the simulation (when all displacing is injected). Note that the total displacement is improved up to 174% comparing with the non-reactive case. The center vertical line indicates the time when the pattern in the non-reactive case reaches the boundary of the domain. The simulation parameters are indicated in Table 1.

Figure 16 shows the total volume of the displacing solution that is recovered at the end of each simulation and the values are compared with the control non-reactive experiment (compare with the experimental Fig. 12).

## Discussion and conclusions

We considered in this contribution a highly viscous polymeric solution and studied the problem of its displacement by another miscible less-viscous solution. We designed the second solution in such a way that a reaction takes place at the interface that locally changes the stability mechanism. In this way, the stable configuration (viscous solution pushing a less viscous solution) becomes unstable and vice versa.

The physical mechanism underlying is a dramatic decrease in the pH at the interface that is induced once the two solutions interact and only at the interface. This change in pH results in two different chemical processes that combined with the fluid displacement are responsible for the destabilization/stabilization of the system: precipitation and the reactive front.

The precipitation is produced by the alteration of the spatial configuration of the polymer at low pH. This process is driven by the protonation of the PAA molecule that facilitates the aggregation and precipitation by hydrogen bond formation. In the direct experiment, the precipitate accumulates in the region between the two solutions and locally reduces the permeability behaving like a stopper. This, consequently, increases the pressure inside the cell. At a certain level of pressure, the crust breaks and the displacing solution is ejected. This break happens by spontaneous symmetry break. In the reverse case, (the less viscous solution displacing a more viscous one), the whole mechanism is the same, just the polymer aggregation process and the flow displacement act synergistically to produce a stable displacement.

The reaction front is produced by a combination of several reaction–diffusion processes. In the first place, the acidic character of the front is explained by the generation of bisulfite at the interface via equilibrium displacement. This reaction is potentiated by the large gradient of protons between solutions A and B.

The Damköhler number (the ratio of the hydrodynamic characteristic time over the temporal scale for the reactive processes involved) is used as an indicator that marks the region of flow rates that can change the stability of the process. Large Damköhler numbers are dominated by significant reactive processes that modify the stability at the interface as the reaction has time to compete with the hydrodynamic process. We also characterized the role of the diffusion against advection and reaction by studying the Péclet and the Péclet-Damköhler numbers, respectively. We demonstrated that differential diffusion is fundamental for the front to occur, and it is mainly produced by the PAA molecule that acts as a proton acceptor. Due to the similarities of the patterns here presented with those observed in dissolution fronts instabilities^42^, other specific numbers, such as Zhao number, may be applied to characterize this system as well^43–45^. However, more research is needed in order to adapt the characteristics of an ideal Hele–Shaw cell system into a more specific application. This will be addressed in future work.

The mechanism observed in the experiments has been translated into a mathematical model and numerically integrated. The results show an important agreement with the experimental evidence demonstrating the validity of the proposed mechanism.

The mechanism used along this manuscript took advantage of the pH-induced precipitation but eventually and depending on the application, different not-involving-precipitation approaches could be imagined. The protocol proposed in this paper opens a path to design reactions that change the stability properties at the interface by synergistic mechanisms between chemical and advective processes. The possible applications are countless in industrial configurations as well as in processes of recovery of natural resources.

## Supplementary Information

Supplementary Video 1.

Supplementary Video 2.

Supplementary Video 3.

Supplementary Video 4.

Supplementary Video 5.

Supplementary Video 6.

Supplementary Video 7.

Supplementary Video 8.

Supplementary Video 9.

Supplementary Video 10.

Supplementary Information.
