# Supplementary material for: A bottom-up approach to construct or deconstruct a fluid instability
Source: Sci Rep. 2021 Dec 21;11:24368. doi: 10.1038/s41598-021-03676-z (PMC8692339; doi:10.1038/s41598-021-03676-z)
Supplement: Supplementary file 11 — Supplementary Information. [file 41598_2021_3676_MOESM11_ESM.pdf]

Supplementary Material

A Bottom-Up Approach to Construct or Deconstruct a Fluid Instability

Darío M. Escala and Alberto P. Muñuzuri*

Institute CRETUS. Group of Nonlinear Physics. Fac. Physics. University of Santiago de Compostela. 15782 Santiago de Compostela, Spain

Supplementary contents

1.- Control experiments.

a.- C1 - Influence of the Color Indicator.

b.- C2 - Influence of the Formaldehyde.

c.- C3 - Influence of the SO_3^{2-}.

d.- C4 - Influence of the PAA.

e.- C5 - Chemical Interaction at the Interface.

2.- Detailed explanation of the instability mechanism.

a.- Crust formation.

b.- Reactive front.

3.- Quantitative measurements of the interface geometrical properties.

4.- Values of circularity.

a.- Direct experiment: Viscous solution displaces a less viscous solution.

b.- Reverse experiment: Less viscous solution displaces viscous solution

5.- Fractal pattern formation in the reverse experiment observed through the Schlieren technique.

6.- Close view during front stabilization in a reverse experiment.

7.- Additional numerical results.

a.- Extended circularity calculations

b.- Pressure field evolution in the direct experiment

8- Supplementary Image analysis.

9.- Reaction velocity and Damhköler number.

10.- Shear-Rate estimation inside the Hele-Shaw cell (γf) and elastic effects.

11.- Diffusion effect, Péclet (Pe), and Péclet-Damhköler (PeDa) numbers estimation.

12.- Mesh independence study.

13.- Description of the supplementary movies.

1.- Control Experiments

Several additional experiments were done following the same procedure explained in the Methods Section but changing the chemical composition of the two solutions in order to determine the role played by the chemical species in the mechanism underlying the instability.

Table S1 summarizes all the control experiments done and complete those presented in the main text. (marked as C6 in the table). The base case corresponds to the standard recipe as described in the methods section.

Many experiments in this section are recorded using the Schlieren technique (described in methods section) [1]. This technique provides detailed information about the diffusive and advective phenomena involved without interference with the color indicator. The observation field in these cases is smaller due to technical constraints.

Here, we put the focus mainly on the direct experiments, where the more viscous solution (solution A) is injected into de Hele-Shaw cell and, thus, displaces the less viscous solution (solution B).

Table S1: Detailed chemical composition of the displacing and displaced solutions used in the control experiments.

CASE	DISPLACING/DISPLACED SOLUTION COMPOSITION						EFFECT
	SOLUTION A					SOLUTION B	
	Form. (M)	Sulfite (M)	PAA (wt%)	C.I. (wt%)	Carbonate (M)	G.A. (m)	
BASE	0.350	0.068	0.438	0.021	0	2	PRECIP./FRONT
C1	0.350	0.068	0.438	0	0	2	PRECIP./FRONT
C2	0	0.068	0.438	0	0	2	PRECIP./FRONT
C3	0	0	0.438	0	0	2	PRECIPITATION
C4	0	0.068	0	0	0	2	----
C5	0	0	0.438	0	0.068	2	CO_2 Formation
C6	0.350	0.068	0.438	0.021	0	0	----

C1 - Influence of the Color Indicator. Figure S1 shows the evolution of the interface when the color indicator in the displacing solution is replaced by doubly distilled water. Note that the instability is produced similarly as in the case with the color indicator (Figures 4-5 and 7 in the main text). This demonstrates that the observed results are not due to an artifact produced by this chemical component. This is also important as many previous works reports that color indicators also behave as proton acceptors like the PAA molecule[2,3]. In our case, the color indicator does not show any significant effect of this nature. Figure S1(b) shows the results

of averaging the experimental frames in a specific region of time (30-200 min, see Figure 7(b)). It is possible to see similar stagnation areas, crust formation, and pattern emergence. The reactive front that competes with the injection flow is also observed (Figure S1(c)).

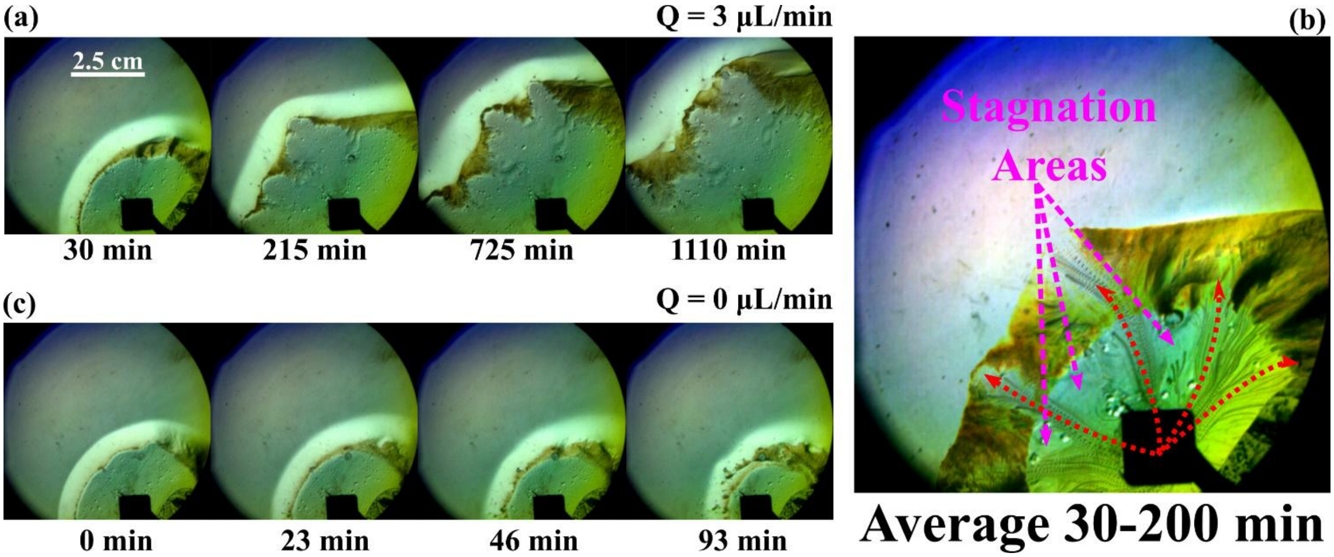

Figure S1: Schlieren images for a control experiment where the color indicator in the displacing solution was replaced by doubly distilled water. The flow rate is 3 μL/min. The system behaves identically to the case with a color indicator. (a) The injection process shows the occurrence of ramifications in the same way as the experiment presented in Fig. 7. (b) Stagnation areas are also observed in the physical process and are involved in the pattern formation. (c) For Q = 0 μL/min the reaction front propagates inwards.

C2 - Influence of the Formaldehyde. Figure S2 shows the effect of replacing the formaldehyde and the color indicator of solution A with doubly distilled water. This experiment is done with a flow rate of Q = 50 μL/min. In this case, it is possible to observe both, the crust formation at the interface and the reaction front once the injection is stopped. This is possible as the chemical timescale is not slow enough to compete with the advective timescale. Same results were obtained at a lower flow rate (experiments are not shown). This demonstrates that the formaldehyde is neither necessary for both chemical processes to occur.

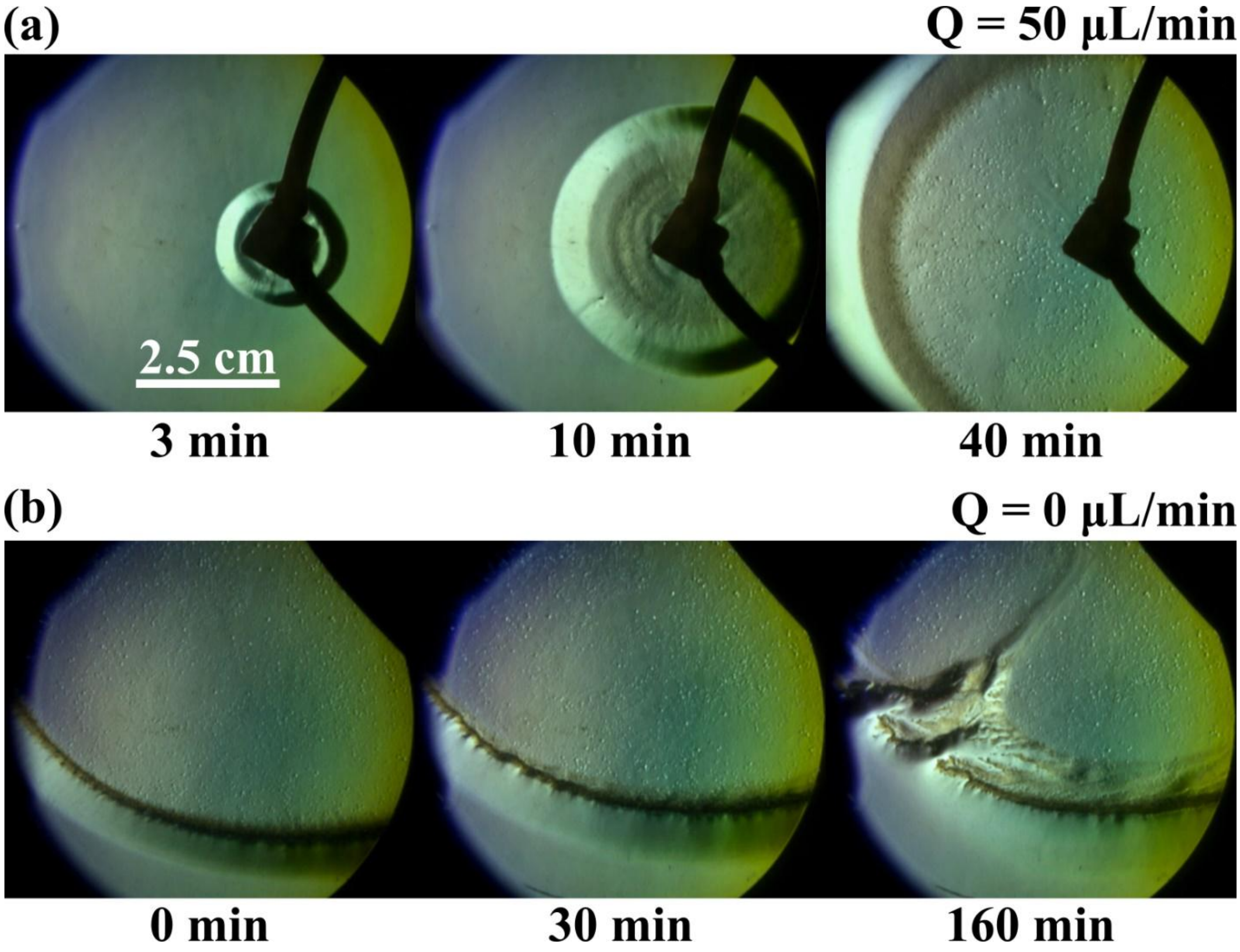

Figure S2: Schlieren images of a control experiment where the formaldehyde was removed from the original recipe and replaced by doubly distilled water. The concentration of the remaining reagents is the same as indicated in the methods section. Q = 50 µL/min. (a) In this case, the advective timescale is faster compared to the chemical timescale, thus the system remains stable. The brownish contour observed at the interface corresponds to the polymer precipitation (this corresponds to the yellow contour in the non-Schlieren experiments presented in the main text). (b) the reaction front is also observed in this system configuration once the injection is stopped.

C3 - Influence of the PAA. This control experiment is carried out by replacing the formaldehyde, the color indicator, and the sulfite with doubly distilled water, letting only the polymer in solution A. Results are shown in Figure S3. As can be seen, the contact between the PAA and the gluconic acid makes the polymer to precipitate. However, no reactive front is observed once the injection is stopped. This experiment suggests that the minimum requirements needed for both chemical processes to occur are the presence of PAA and the SO_3^{-2} as shown in Figure S2. As there is no competitive reaction front, the system remains stable during the entire experimental run.

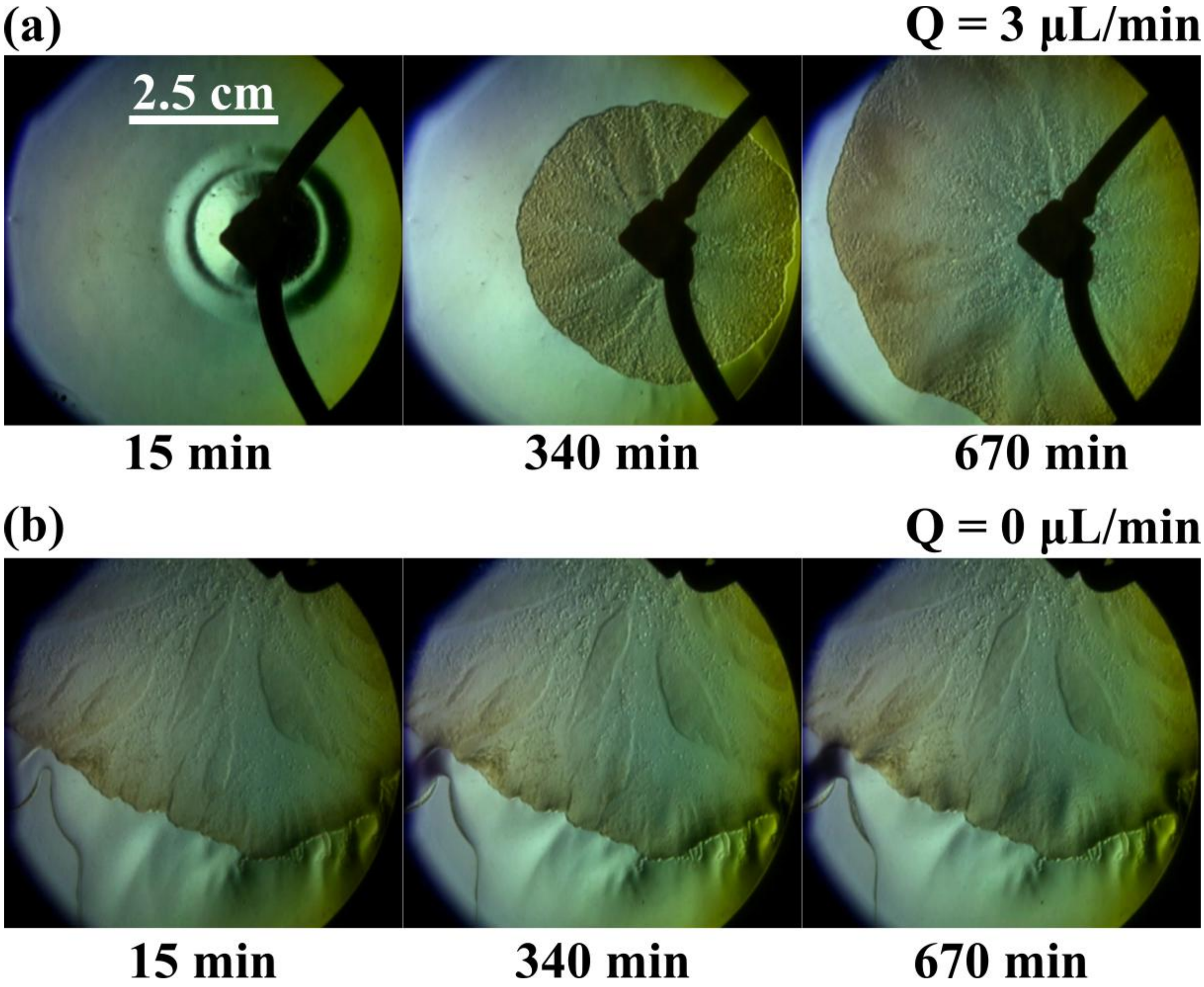

Figure S3: Control experiment where the displacing solution is only composed of PAA in the same concentration as the base case indicated in the Methods section and the remaining reagents are replaced by doubly distilled water. The displaced solution remains unaltered concerning the original formulation. (a) the interaction between the polymer and the gluconic acid makes the polymer to precipitate. However, no pattern formation is observed, and the system remains mostly stable. (b) No chemical front is observed when the injection is stopped.

C4 - Influence of the SO_3^{2-}. In this case, solution A is only composed of SO_3^{2-} with the same concentration as the base formulation replacing the remaining reagents with doubly distilled water. This experiment is intended to show the major role played by the polymer in both reactive processes. As can be seen in Figure S4, neither precipitate nor chemical front is observed when sulfite is only injected into the medium. This result is expected based on the results obtained in the previous control experiments.

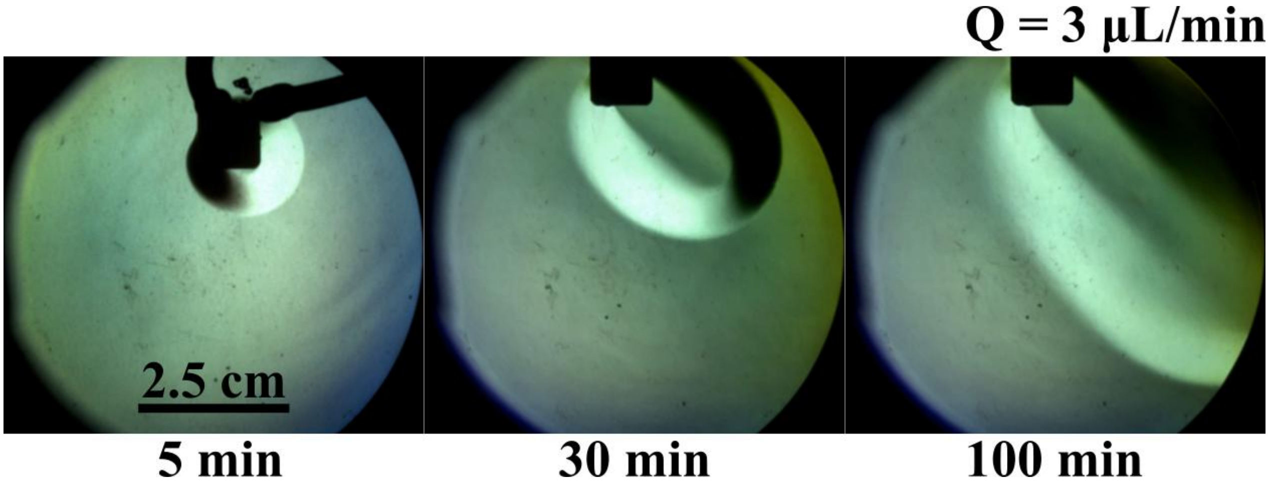

Figure S4: Control experiment where the displacing solution is composed of SO_3^{2-} in the same concentration as the base case indicated in the methods section. The displaced solution remains unaltered with respect to the original formulation. As can be seen, neither precipitation nor chemical fronts are observed. As both solutions are miscible liquids with almost identical viscosity ($\mu \approx 1$ mPa s), the interface grows deformed.

C5 - Chemical Interaction at the Interface: The last control experiment is intended to show the chemical interaction between displacing and displaced solution at the contact interface. In this case, the displacing solution is composed of PAA and the SO_3^{2-} is replaced by the same concentration of CO_3^{2-}. The remaining components of the base formulation are replaced by doubly distilled water. The carbonate concentration used in this experiment is not detrimental to the polymer rheology. Also, the viscosity of the solution is similar to the viscosity in the base case.

The goal of this experiment is to demonstrate the equilibrium displacement mechanism at the interface between both fluids. At the same time, it works as a secondary test to verify the major importance of the sulfite in the reactive mixture. Results are presented in Figure S5. Some crust accumulation is observed at the interface. However, neither pattern formation nor reactive front is appreciated. Moreover, several CO_2 bubbles emerge from the interface between both liquids once the displacing is injected. These bubbles grow during the entire experimental time.

Similarly to the sulfite, the carbonate has many equilibriums in solution. Even though the interaction of both molecules with the polymer is completely different, the presence of CO_2 bubbles suggests that the interfacial condition is acid enough to displace part of the carbonate equilibrium into CO_2 as indicated by the equilibrium equations,

$$CO_3^{2-} \leftrightarrow HCO_3^{-} \leftrightarrow H_2O.CO_2 + CO_2\,(g)$$

The equilibrium is displaced to the right side for pH < 6[4]. As previously indicated in the manuscript, the gluconic acid solution has a pH ≈ 2. This agrees with the obtained results.

For the sulfite, the equilibrium equations are similar,

$$SO_3^{2-} \leftrightarrow HSO_3^- \leftrightarrow H_2O.SO_2$$

However, at pH ≈ 2, most of the sulfite in the solution is in the form of bisulfite[5]. This demonstrates the presence of bisulfite in the medium and agrees with our mechanism proposal.

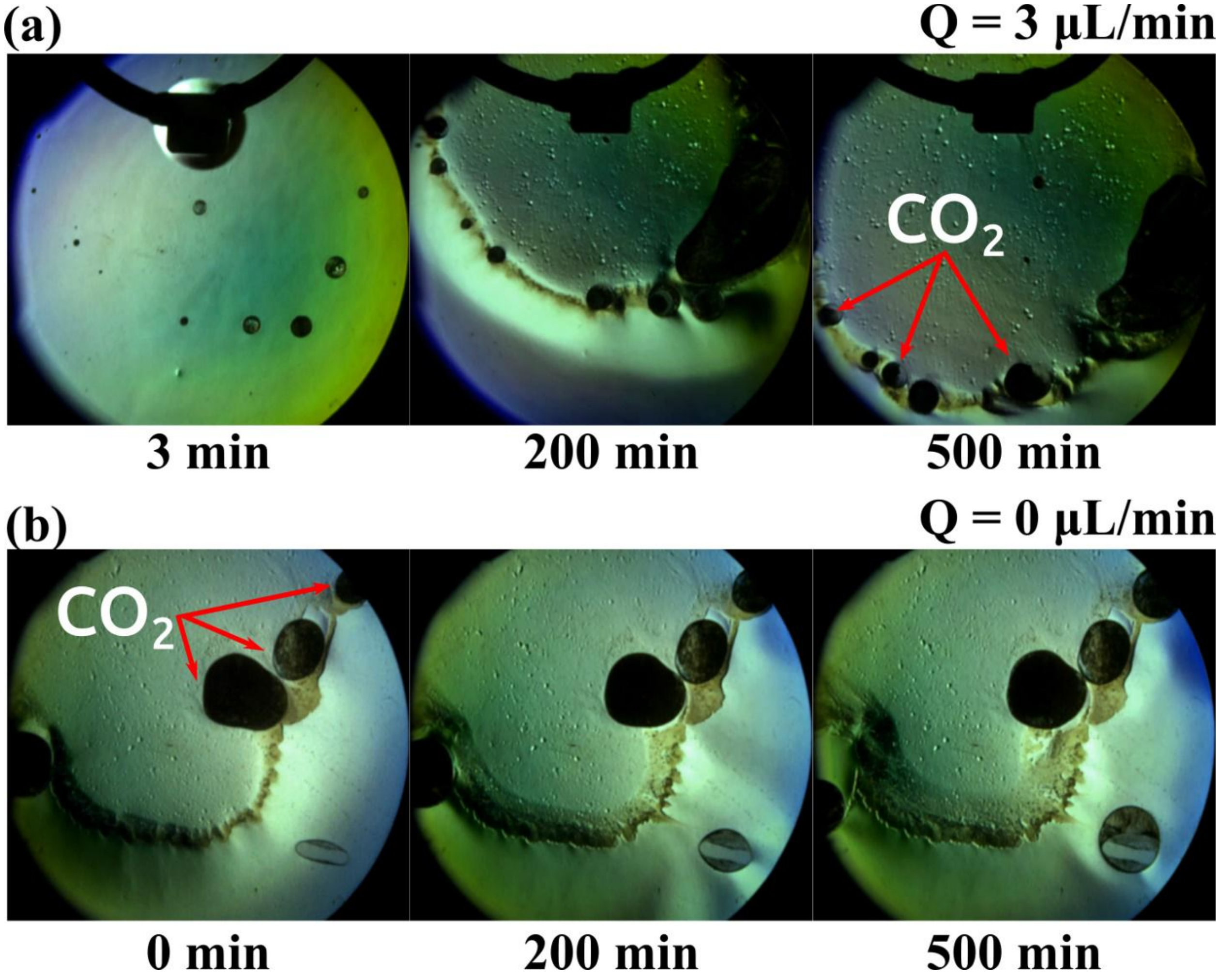

Figure S5: Control experiment where the displacing solution is composed of PAA and CO_3^{2-}. The concentration of PAA remains the same as the original formulation. The carbonate concentration is the same as the sulfite concentration indicated in the methods section. The displaced solution composition remains unaltered. (a) Both precipitation and bubble formation are observed when the injection starts. The bubbles grow during the entire experimental run. (b) Once the injection is stopped, no chemical front is observed. However, the bubbles continue to grow. These bubbles indicate the presence of CO_2 in the cell, produced by the acidic condition at the interface.

2.- Detailed explanation of the instability mechanism.

Using all the information obtained from the control experiments, and the experiments reported in the main text, it is now possible to give a more detailed explanation of the instability mechanism.

a.- Crust Formation: From control results C1-C3, it is possible to infer that the presence of PAA is fundamental for the crust formation (no crust is observed without PAA in the medium). Moreover, a low pH condition is needed, as no effect is observed when solution B is replaced by doubly distilled water (C6). This condition is provided by gluconic acid. As previously explained, the PAA molecule is affected by the pH of the medium[6–8]. At higher pH, the carboxylic groups of the PAA molecule dissociate changing into carboxylate ion. The negative charges of the carboxylate groups produce a repulsive effect that elongates the molecule into a rodlike type structure that critically increases the viscosity of the solution. In contrast, at lower pH, both intra- and intermolecular hydrogen bonds compact not only the PAA structure but also produce molecule agglomeration. This agglomeration facilitates the precipitation by the large side chain of the PAA molecule (in our case 4000000 MW). Precipitation is also observed in an assay tube when different acid solutions are added to PAA formulations. In particular, we analyzed mixtures of PAA solutions with gluconic acid (solution B) and with HSO_3^- 0.0684 M. All mixtures are centrifuged using a Hettich Universal 320 R centrifuge for 5 minutes at 5000 rpm at 23 °C. Results are shown in Figure S6.

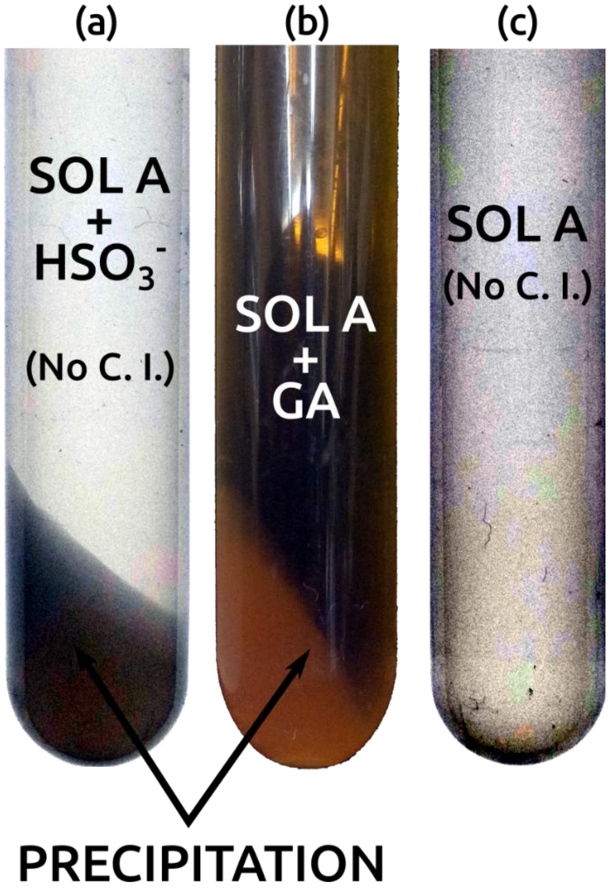

Figure S6: Precipitation test performed for three different preparations: (a) a mixture containing 50 % solution A (without color indicator), and 50% 0.0684 M of HSO_3^- (b) mixture containing 50% solution A and 50% solution B (Gluconic acid 1.66 M) and (c) only solution A (without color indicator). All assay tubes were centrifuged for 5 minutes at 5000 rpm at 23 °C. Precipitation was only observed for (a) and (b) cases.

This experiment demonstrates that precipitation is observed when a PAA solution is exposed to an acidic condition. The original mixture itself (solution A) does not show any precipitation. This agrees with the fact that no precipitation was observed when the gluconic acid was replaced by doubly distilled water (experiment C6) and demonstrates the stability of the solution.

In physical terms, the precipitation affects the fluid displacement by decreasing the permeability of the porous matrix locally. Several works reported the influence of such phenomena in the generation of hydrodynamic instabilities[9–11]. The permeability loss increases the pressure of the system. This increment in pressure is responsible for the ejection of the displacing solution that produces the characteristic shape of the instability observed in the direct case.

b.- Reactive Front: From experiment C3 it is possible to observe that the simplest system where the reactive front is observed is when solution A is composed of PAA and SO_3^{2-} and solution B is gluconic acid (see Table S1). Previous works demonstrates that HSO_3^- is generated by equilibrium displacement if SO_3^{2-} is added to a PAA aqueous solution. This process is mediated by the polymer dissociation in H_2O as following[12,13]:

$$PAA \leftrightarrow PA^- + H^+$$

$$SO_3^{2-} + H^+ \leftrightarrow HSO_3^-$$

where PA^- is the polycarboxylate ion.

For the reaction front to occur, gluconic acid must be present in solution B. As described in Kovacs *et al*[14,15], the gluconic acid is dissociated in an aqueous solution as following:

$$GA \leftrightarrow G^- + H^+$$

where GA is the Gluconic Acid and G^- is the gluconate ion. The pH of solution B is about 2.

In the first place, the PAA is well known to be a reversible proton acceptor[2,3]. This molecule affects the apparent diffusivity of protons and was extensively used to modify the dynamics of classical pH-oscillators where autocatalytic fronts are common[2]. However, this chemical front cannot be produced by an autocatalytic process as there are no oxidant species in the medium. Thus, this suggests that the nature of this chemical front is different from the classical autocatalytic approach.

In the second place, C5 shows that when sulfite is replaced by carbonate, CO_2 bubbles emerge from the interface between solution A and B. This indicates that the acidic condition is strong enough to displace the carbonate equilibrium from CO_3^{2-} into CO_2. This also suggests that when SO_3^{2-} is in the mixture, the same displacement occurs to the sulfite equilibrium producing bisulfite, which is essentially an acid species. This result explains why the chemical front "converts" the blue coloration (basic) into a yellowish coloration (acid) when the base formulation of solution A is used. (This is not observed if SO_3^{2-} is not present in the mixture).

Finally, there is also a large difference in diffusivity between the PAA and H^+ of solution B. Many works reported that the diffusion coefficient of the PAA molecules is at least two orders of magnitude less compared to the water diffusivity (D_{PAA} = 1 x 10^{-11} m^2/s)[7,12,16]. It is also known that protons diffuse faster than water (D_{H^+} = 9.3 x 10^{-9} m^2/s) [17,18]. The chemical front is then a combination of all the reaction-diffusion processes described.

In physical terms, the front stabilization on the reverse experiment is obtained when the flow timescale is slow enough to allow the reaction to occur. In this case, the chemical front moves aligned to the flow and reduces the pH of the medium. This facilitates the polymer precipitation. However, here the polymer does not block the flow and work as an effective wall that drags the more viscous fluid. When the flow timescale is large, the reaction is slower, and classical viscous fingering is observed. Even though there is an unstable hydrodynamic configuration between the low viscous displacing solution and the polymer wall, the overall process is stable (see Figure S11).

3.- Quantitative measurements of the interface geometrical properties.

Some other observables were used in the literature[19,20] to describe the shape of similar patterns such as density area or fractal dimension. In our experimental case, we found that the circularity provides a more accurate description due to the nature of our result. Nevertheless, the use of circularity is justified based on previous works where results obtained by using both tools, were comparable[12]. Here we consider another parameter that can also be used to characterize our experiments. Analogously to the circularity, the change in the injection front is also described analytically by measuring the average advancement of the displacing solution over time and comparing both, reactive and non-reactive cases in a relative timescale. This analysis is also useful to evaluate the reaction influence in the observed patterns. The results are plotted in Figure S7 for three different inflow velocities. In the vertical axis, we plot what we called the average displacing profile. This quantity results from determining the interface location and then measuring the distance from every point at the interface to the center of the cell. Figure S7a shows the average of these measurements that directly gives the average displacing profile and the standard deviation providing a measurement of how irregular is the interface in the reactive case. Thus, circular interfaces are characterized by an almost negligible deviation while for low flow rates and instability at the interface the dispersion must be very large. Figure S7b is a plot of the average displacing profile for the same control experiments and its evolution with time. As expected, the dispersion of the profile is small and constant.

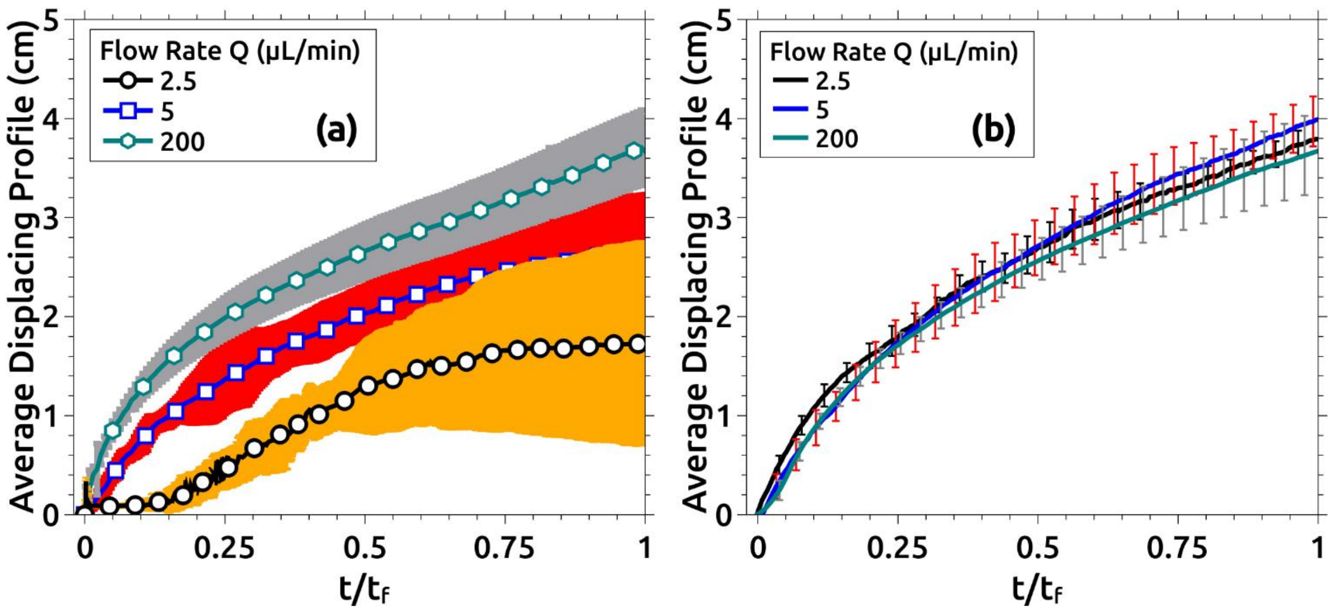

Figure S7: Quantitative comparison of the displacing solution profiles in a relative timescale for (a) reactive and (b) control experiments. All results are scaled to the final time for each experiment (t_f) as described in the main text. Results are presented as the average (marked lines) and the colored areas correspond with the range of distances to the cell center from each point at the interface. Marks are intended as a visual guide. For higher Q (200 μL/min), the profile describes the typical circular front. The dispersion, in this case, is well-bounded as can be observed in Fig. 5. As the flow rate is decreased (5 μL/min), the reaction processes become more important producing changes in the average profile and increasing the dispersion. For the lowest flow rate analyzed (2.5 μL/min), the dispersion is maximized, especially for $t/t_f > 0.5$. These results are coherent with the experimental observations presented in Fig. 5 and the results of Fig. 6. This demonstrates that without reaction influence the circularity of the interface remains stable and almost unaltered during all the experimental run.

4.- Values of circularity.

a.- Direct experiment: Viscous solution displaces a less viscous solution

Figure S8a shows the evolution of the circularity for all the flow rates considered. Note that low flow rates produce low values of the circularity as the interface gets distorted. Large flow rates produce no instability and, thus, the interface remains circular and circularity is almost one. Intermediate cases are characterized by a circularity close to one in the first stages and this value drops to lower values as the actual velocity at the interface becomes smaller along with the experiment. Figure S8b shows the circularity for the control experiments where no instability is observed. In these cases, the circularity value remains close to one and constant along with the experiment.

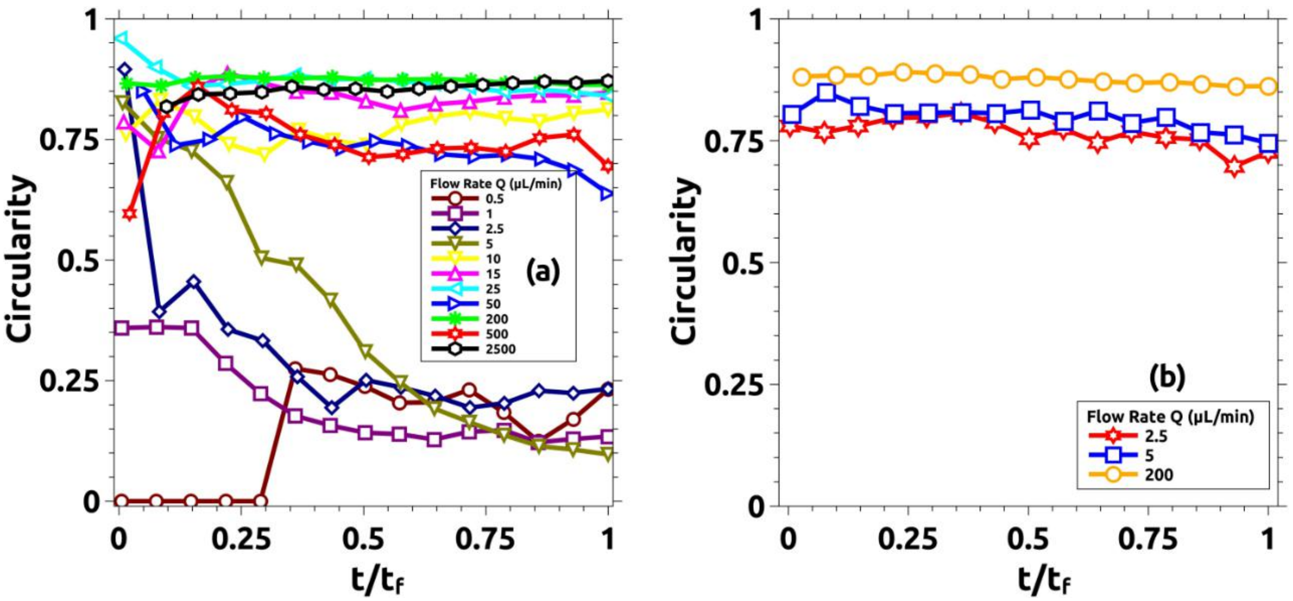

Figure S8: Circularity vs normalized time t/t_f for all the (a) reactive and (b) non-reactive (control) experiments. This plot complements those presented in Figure 6. The results for the control experiment are coherent with the results presented in the manuscript. The circularity remains close to 1, indicating that such fronts remain mostly circular during the experimental realization even at low flow rates.

b.- Reverse experiments: Less viscous solution displaces viscous solution

Figure S9a shows the circularity values for the reverse experiment case for all the flow rates considered. Lower circularities are obtained for higher flow rates. This is expected, as the system gets more hydrodynamically unstable as the flow rate is increased. However, the circularity does not remain constant and increases in time due to reactive effects (see Figure 11 of the main manuscript). For Q = 10 μL/min, the system stabilization is reflected by an almost constant value of the circularity. For the non-reactive case (Figure S9b), the circularity drops faster and no variation is observed during the experimental run.

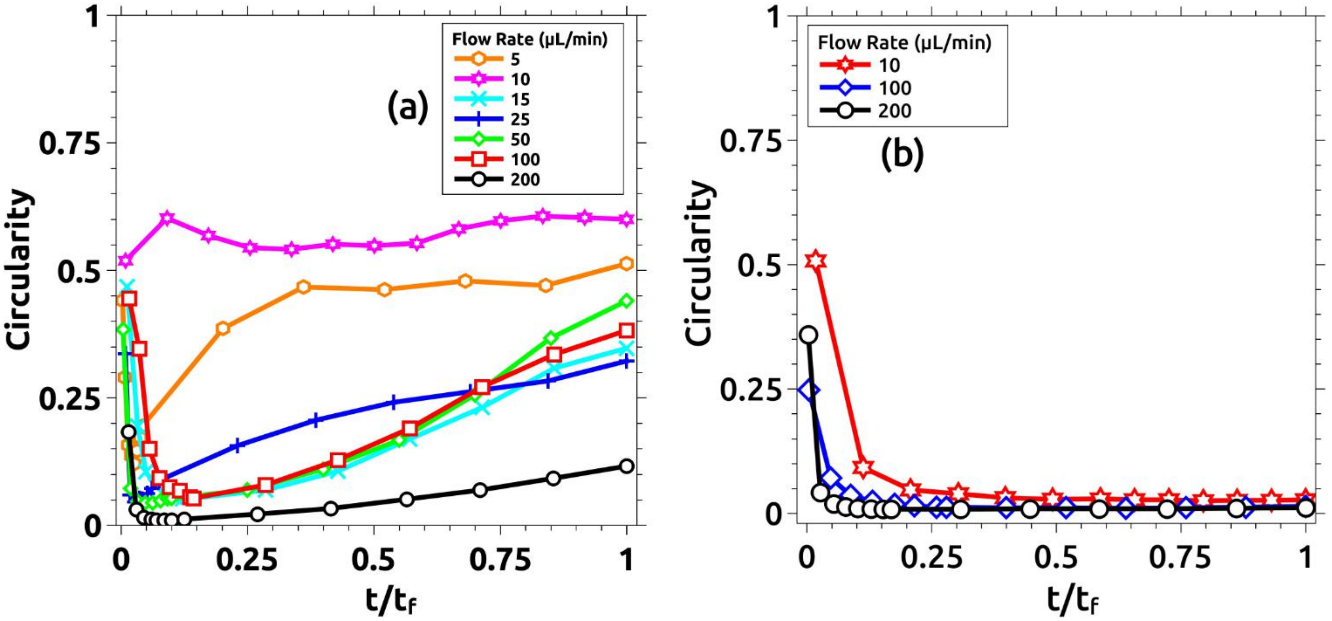

Figure S9: Circularity vs t/t_f for all the (a) reactive and (b) non-reactive experiments in the reverse case. These plots complement those presented in Figure 11. The circularity of the control cases remains close to 0, indicating a pure fractal structure very far from a circular shape during the entire experimental run. In this case, diffusive effects are negligible. In the reactive cases, as the flow rate is reduced, the systems became more stable, and thus, the circularity closer to 1.

5.- Observation of fractal pattern formation in the reverse experiment observed with the Schlieren technique.

Figure S10 shows how the fractal pattern is almost instantly generated due to the differences in viscosity between displacing and displaced solutions. This behavior continues until the displacing solution reaches the cell boundary (approximately 1.5 min after the beginning of the experiment). 15 minutes later, it is possible to appreciate how the reaction produces the polymer aggregation (or crust). The process increases the finger thickness and consequently, the circularity is also increased as shown in Figures 9-11.

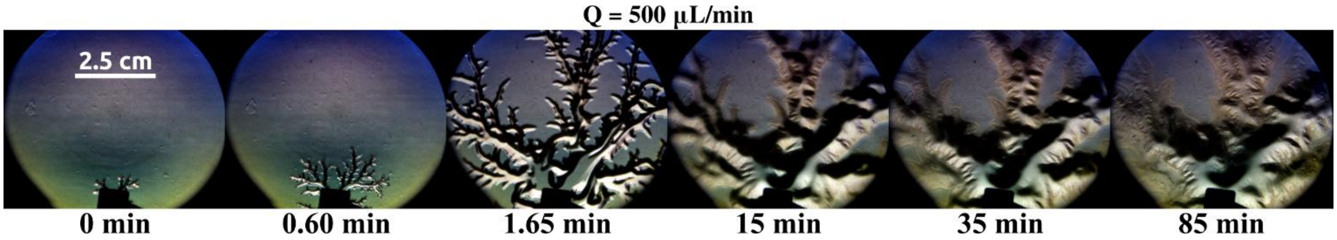

Figure S10: Example of an unstable situation in the reverse experiment observed through the Schlieren optics. Here, the velocity of the flow is faster compared to the polymer aggregation, thus, the system remains unstable. Fractal patterns are observed to propagates quickly towards solution A until the displacing fluid reaches the reactor boundary. The finger thickness changes in time due to chemical interactions. This effect is visible for t > 15 min.

6.- Close view during front stabilization in a reverse experiment.

For low values of the flow rate, a less viscous solution can effectively displace a more viscous one (reverse experiment). The physical details of this mechanism are observed in Figure S11 for Q = 10 µL/min.

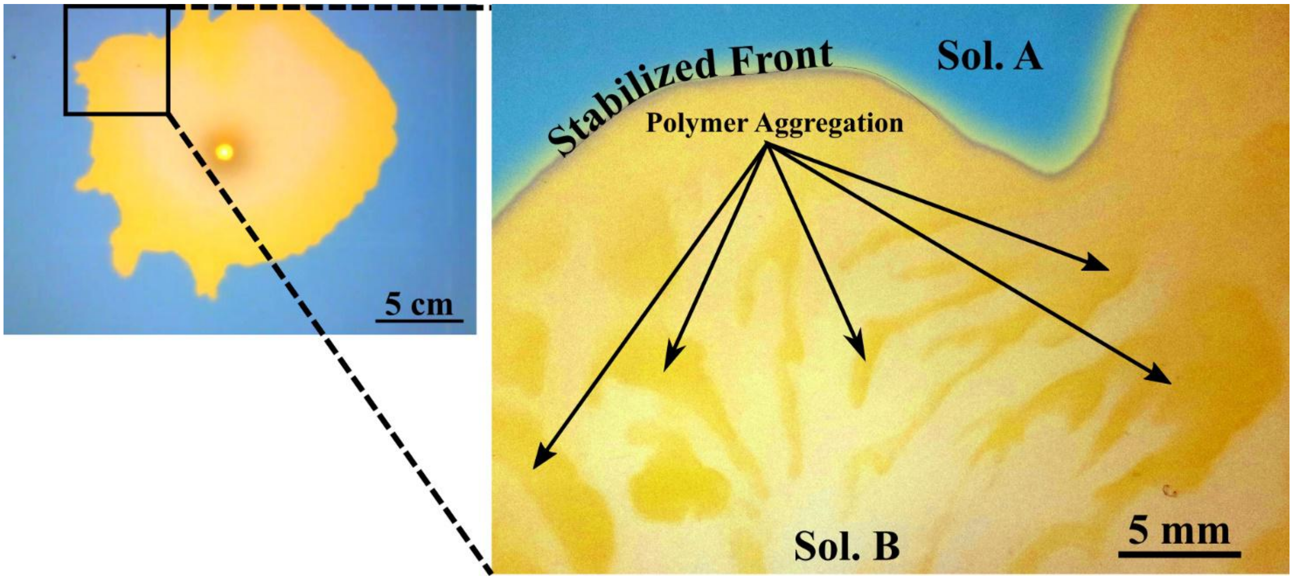

Figure S11: Close view of the front stabilization in the reverse experiment. This image illustrates the physical mechanism involved in the stabilization in a viscous fingering situation for the case where Q = 10 µL/min. Similarly to the results presented in Figure 13, when the flow is slow enough, the reaction produces a polymer aggregation wall, making possible the drag of solution A. The polymer aggregation is appreciated as a brownish crust as the one observed in Figure 7 in the direct experiments. Some patterns are locally produced between both solutions due to the polymer precipitation. However, the competition between the reaction rate and the displacing flow makes the system globally stable.

7.- Additional numerical results.

a.- Extended circularity calculations.

Figures S12-S13 show an extended examination of the simulation results. Similar to Figures S8-S9, Figure S12 shows the circularity calculation for all the studied cases. In Fig. S12a, the circularity is calculated for the direct experiment simulation in both, reactive (left panel) and non-reactive (right panel), cases. As can be observed, the model reproduces well the drastic change in the circularity produced by changing the flow rate. For larger flow rates, the circularity remains closer to 1 as the reaction timescales are slower compared to the advective timescales. For the non-reactive case, the circularity is always closer to 1 as there is no reaction involved in the pattern generation. Figure S12b shows the same analysis for the reverse experiment. The simulation also shows good agreement with the experimental results presented in Figure S9. For higher flow rates, the circularity remains closer to 0 as the system is hydrodynamically unstable. For lower flow rates, both reaction and advection timescales work synergistically stabilizing the system. The increment in circularity in time observed for the largest flow rates are also well reproduced by the model. In the non-reactive case, the circularity remained closer to 0 as the system is always unstable.

Figure S13 shows the effect of the flow rate in pattern formation. Analogously to Figures 5 and 10 of the main text, Figure S13 shows how the pattern formation is affected by changing the flow rate for the direct (Fig.

S13a) and the reverse (Fig S13b) experiments at the same normalized time (t/t_f = 0.9). For the direct case, the concentration fields of A and C were overlaid for facilitating observation of the polymer precipitation (crust wall).

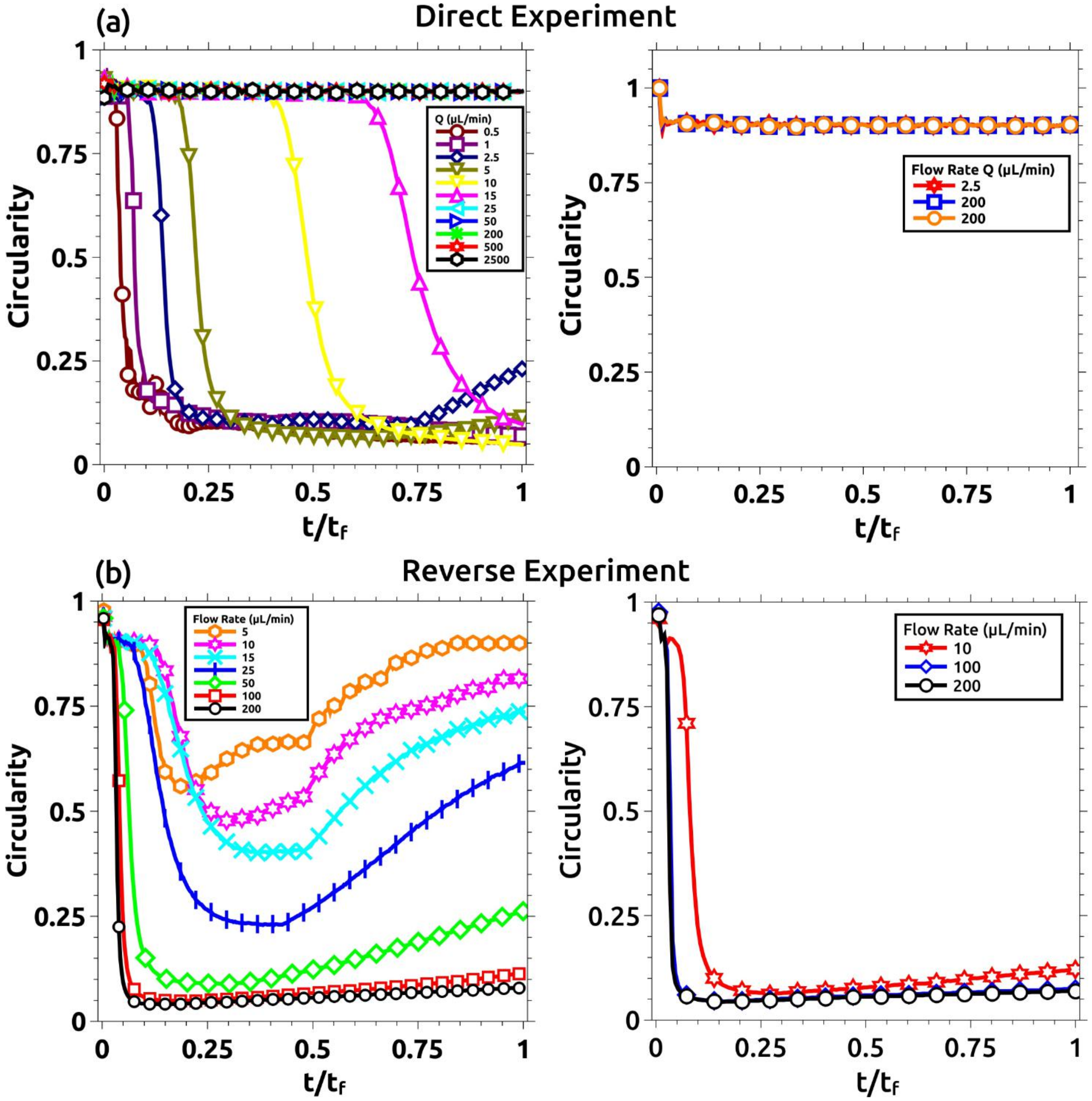

Figure S12: Numerical circularity variation as a function of the flow rate Q for all the simulated cases. (a) Circularity calculation of the direct experiment for the reactive (left panel) and non-reactive (right panel) cases. (b) circularity calculation of the reverse experiment for the reactive (left panel) and non-reactive (right panel) cases. All results are presented in normalized time (t/t_f). All simulation parameters and conditions are indicated in Table 1 of the manuscript.

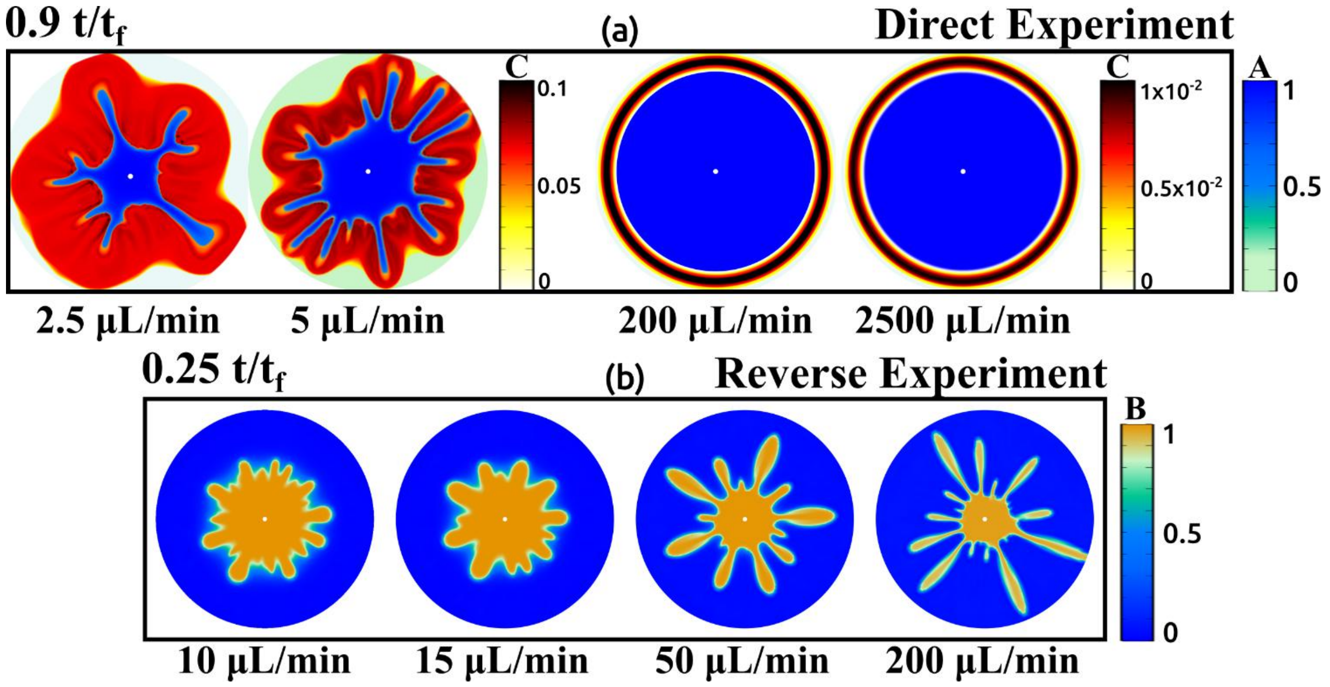

Figure S13: Influence of the flow rate in the pattern formation. Similar to Figures 5 y 10 of the main text, the effect of changing the flow rate in the pattern formation is presented for (a) direct experiment and (b) reverse experiment. Snapshots are taken at the same normalized times as the experiments. In (a), the concentration fields of A and C are overlayered and shown scaled for better comparison with the experimental cases. The simulation parameters are indicated in Table 1 of the manuscript.

b.- Pressure field evolution in the direct experiment.

All species in the simulations are treated as interpenetrating continua. As no solid phase is considered, neither the crust nor the cracks observed experimentally are formally modeled. Motivated by previous work of Shukla et al[10], the precipitate is modeled as another chemical species in liquid phase. Due to Darcy's Law, the pressure gradient is inversely proportional to the permeability. In our model, the permeability is affected by the precipitate concentration, which produces variations in the pressure field. Even though the cracks in the precipitate wall are not included in our simple model, the pressure field changes accordingly to what we observe in the experiments.

Figure S14 show line profiles of the normalized pressure over the numerical domain as a function of time for different cases on the Direct Experiment. As can be seen, even though the cracks are not formally included in our model, the pressure behaves as expected. For the lower flowrates (2.5, 5, and 10 μL/min), the pressure increases until a local maximum that occurs just after pattern formation (blue dashed circles). For the fastest case (50 μL/min), the pressure increases constantly until the displacing solution reaches the outer boundary (purple dashed circle). The snapshots located below the plots correspond with the concentration field of solution A and the normalized pressure at the times where the local maximum occurs.

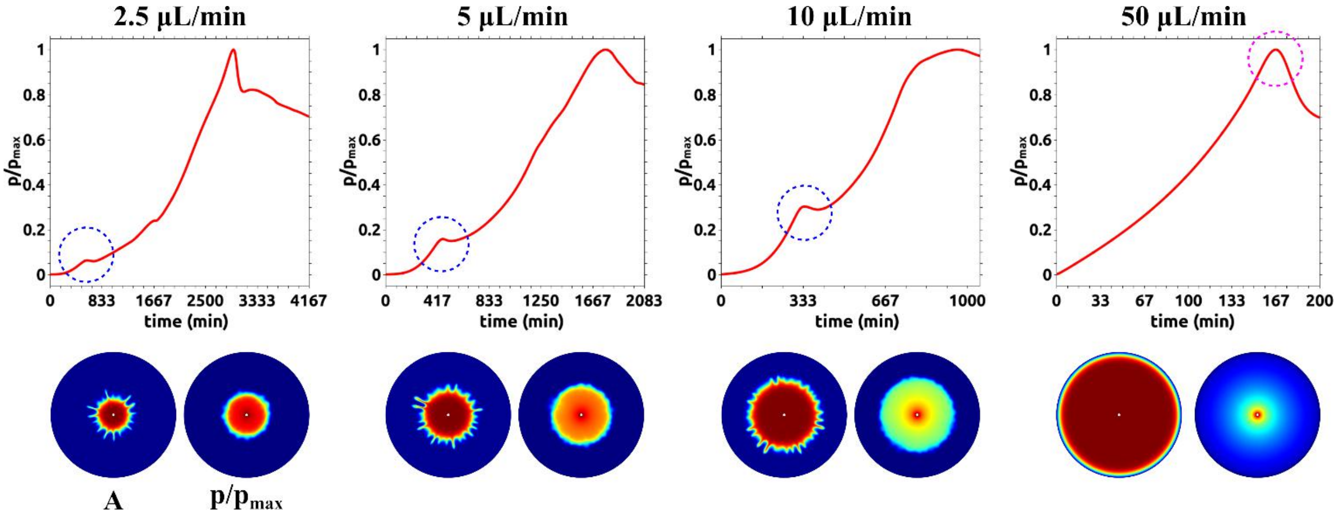

Figure S14: Plot profiles of the normalized pressure field at different flow rates (2.5, 5, 10, and 50 μL/min) for the direct experiment. For the lower flow rates, the pressure increases until a local maximum produced just before pattern formation. Once the system becomes unstable, the pressure field locally reduces. The pressure continues to increase until the displacing fluid reaches the outer boundary. No local maximum is observed for the faster case (50 μl/min).

As can be observed, a temporary decrement in pressure is produced when the system becomes unstable. This phenomenon corresponds to the moment where the crust cracks in the experiment. Moreover, it is also possible to see in these cases how the pressure field is not uniformly distributed in the space delimited by the displacing fluid and the precipitate.

On the other hand, no local maximum is observed in the faster case (50 μL/min), in which the maximum pressure is always located at the inlet boundary and the front remains stable during the entire simulation (purple dashed circle). The pressure decreases only when the displacing fluid reaches the outer boundary.

8.- Supplementary Image analysis.

The methodology to obtain the average displacing interface for the direct experiments shares some similarities with the procedure used to calculate the circularity (Figure 2 of the manuscript). Several space-time plots (STP, indicated as P_1, P_2… P_n) are obtained by taking radial slices of a complete experimental run. These STPs are processed by using the same H&E color deconvolution algorithm used in Fig. 2, obtaining three color components for each set of STPs. Color component 1 is then selected and binarized (this component showed the best results for the profile characterization). The obtained set of binary STP's is then processed by edge detection and binary skeletonization algorithms that convert the image data into a set of numerical curves. From each set of numerical profiles, an average displacement and its dispersion (standard deviation) are calculated as presented in Fig S15.

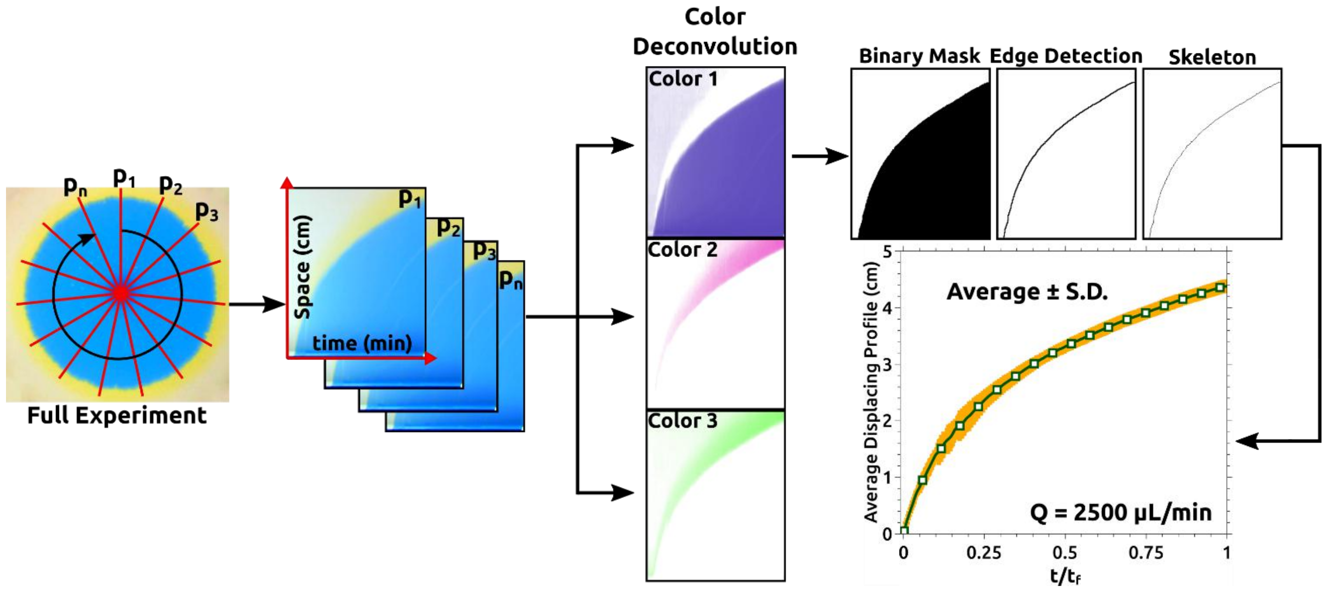

Figure S15: Calculating the average displacing profile for the direct experiment. In this case, a stack of n-space-time plots is obtained from an experiment by performing a radial resliced operation as indicated in the figure. Every frame of such stack is then filtered by a color deconvolution algorithm obtaining three stacks, one for each color component. The binary mask obtained from color 1 is then processed by an edge detection algorithm obtaining a well-defined profile function. A skeletonize algorithm is finally used to retrieve the space and time coordinates used for statistical calculations.

9.- Reaction velocity and Damhköler number.

The Damhköler number (Da) is a non-dimensional number that compares the reactive processes with the hydrodynamics. Following previous works [19,21–23], we calculate Da in a radial Hele-Shaw cell as,

$$Da\,(R) \;=\; \frac{v_r\,R^2}{2\,Q}$$

where v_r is the velocity of the reaction involved and Q is the volumetric flow.

The value of v_r was calculated directly from the Hele-Shaw cell by measuring the reactive front observed once the injection of the displacing solution is stopped. This reactive front propagates from solution B towards solution A. Figure S16a shows four different snapshots of this process. Taking several radial profiles from the injection hole through the cell (black dashed lines in Figure S16a), a Space-Time Plot (STP) like the one presented in Figure S16b is obtained. The reaction velocity v_r is then calculated by measuring the slope of the STP as indicated. The average value for v_r, obtained over several experiments is,

$$v_r\,[mm/min] = \;0.047\;\pm\;0.006$$

for a constant separation gap h = 0.25 mm.

Note that as the radial velocity of the interface is variable in a radial Hele-Show cell such as the one used for our experiments, the Damhköler number will be variable too. Note that this value is far larger than the diffusion velocities in the system. The Péclet number (Pe) and the shear rate (γ_r) inside the cell were also estimated to study the effect of diffusive processes and elasticity in the pattern formation. Details are given in the SI.

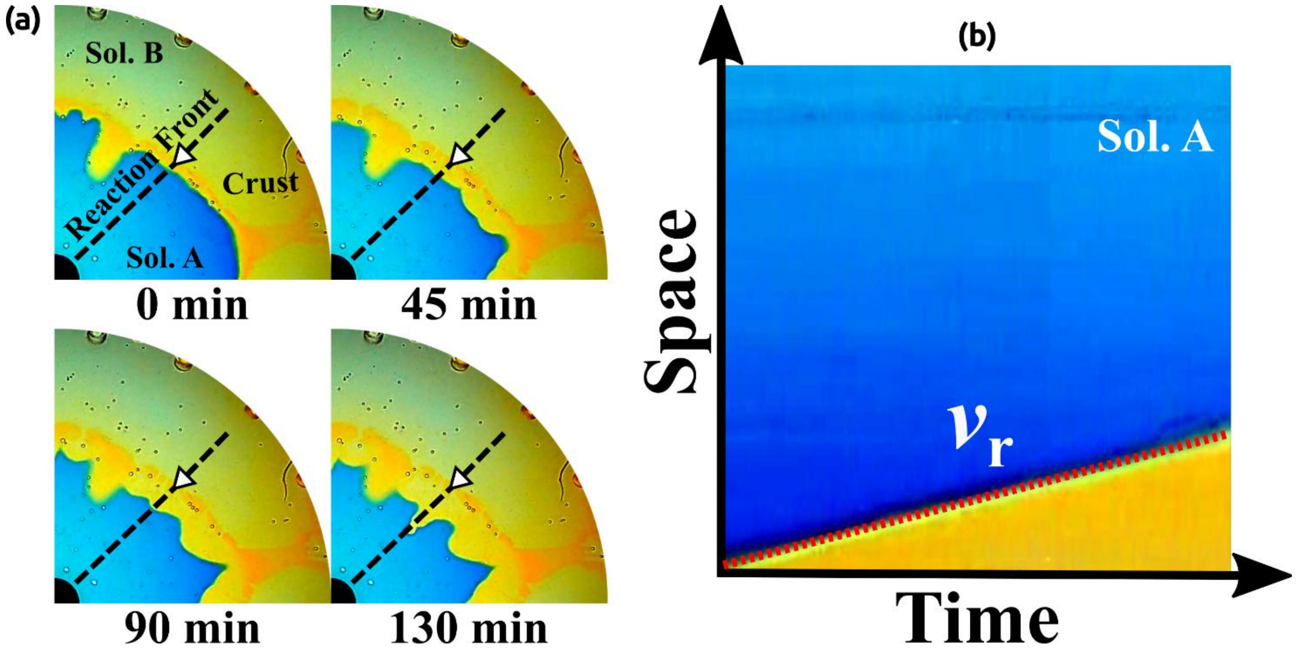

Figure S16: Measuring the reaction front velocity used to estimate the Damhköler number. (a) once the injection of the displacing solution is stopped, a reactive front is observed to move against the flow direction. This reactive front competes with the advancement of solution A. (b) The front velocity v_r is estimated by measuring the slope of the Space-Time plot obtained by taking radial profiles of the cell.

10.- Shear-Rate estimation inside the Hele-Shaw cell (γ_f) and elastic effects.

The estimation of the shear rate is important to discard any elastic effect of the polymer solution inside the Hele-Shaw cell. In previous works [9,13], we demonstrated, for fluids with similar compositions, the absence of elastic effects for the range of flow rates analyzed. In this particular case, we estimated $\boldsymbol{\gamma_f}$ at the injection hole (where the shear rate is maximum) following previous works [19–23] as,

$$\boldsymbol{\gamma_f} = \frac{Q}{\pi R_i h^2}$$

where Q is the volumetric flow rate, *h* is the separation gap and R_i is the radius of the cell. As explained in the aforementioned references, the shear rate is maximum at the injection hole. Thus, in our case $\boldsymbol{\gamma_f}$ is limited to the following range,

$$0.021 < \dot{\gamma}_f\ [s^{-1}] < 106.103$$

for 0.5 < Q [μL/min] < 2500 respectively.

As an additional analysis, Figure S17 shows the first normal stress difference (N_1) of solution A, measured by using a TA-AR2000 rheometer[12]. Results are compared with a reference solution 0.5 wt% PAA (4 x 10^6 MW) + 0.023 M NaOH with relatively strong elasticity. This measurement is useful to discard any artifact associated with the elasticity of the polymer in the cell[20,23]. No elastic effects are observed in the range of shear rates studied for solution A.

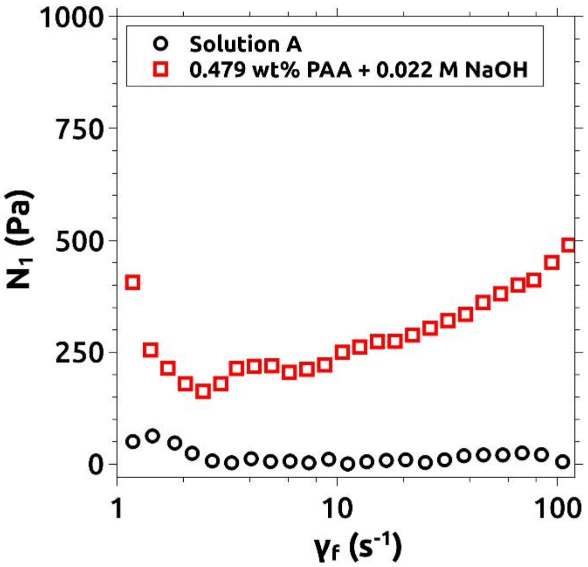

Figure S17: First Normal Stress Difference (N1) measured for solution A (black circles) and a high elasticity reference solution composed of 0.479 wt% PAA and 0.025 M NaOH (red squares). As can be observed, no elastic effects are appreciated for this solution in the range of maximum shear rates studied.

11.- Diffusion effect, Péclet (Pe), and Péclet-Damhköler (PeDa) numbers estimation.

The Péclet number, that describes the relationship between the diffusive and the advective processes, is calculated based on the protocol described previously [19–23]. For a radial Hele-Shaw cell, this number can be calculated as,

$$Pe = \frac{Q}{2\pi b D}$$

where Q is the volumetric flow rate, b is the separation gap between the two cell plates and D is the diffusion coefficient between the more- and less-viscous fluids.

Following Nagatsu *et al* [21], D can be estimated to be 1 x 10^{-9} m^2 s^{-1} and represents the average diffusion coefficient between the polymer and protons at small concentrations in water. Based on this information, the Péclet number is estimated for the lowest and highest flow rates used giving the following values:

$$5.30 < Pe < 26525.82$$

for 0.5 < Q [μL/min] < 2500 respectively.

These results indicate that the advective process dominates over diffusion independently of the flow rates, even for the most extreme cases where the Q is extremely slow. However, as in our system diffusion also plays a major role in the nature of the chemical front, it is also important to analyze the relationship between the diffusive and reactive processes. The Péclet-Damkhöler number (PeDa) is an effective measure to understand the influence of such processes in the development of pattern formation. For this case, the PeDa number is calculated as,

$$PeDa(R) = \frac{v_r R^2}{4\pi b D}$$

where v_r is the reaction front velocity, R is the cell radius, b is the separation gap and D is the diffusion coefficient. We estimate the PeDa number considering the diffusion coefficient of the proton and the H_2O. Larger PeDa numbers indicate that the reaction processes are predominant to diffusion processes. In our case, we want to demonstrate that the fast-diffusive action of the protons provided by the gluconic acid plays a major role in the pattern formation, more specifically, in the stabilization process observed in the reverse case.

We consider a hypothetic case where a reaction occurs, but solution B has a diffusion coefficient similar to water (1 x 10^{-9} m^2/s). As can be seen in Figure S18, the PeDa number is always above 1. This indicates that the reaction effects are more important than diffusion effects. When B diffuses similarly to protons, the PeDa number is below 1 for R < 6 mm. This indicates that for such a radius, the diffusion processes are predominant to the reaction processes.

This can be observed in the simulations, and the diffusive effects are particularly noticeable analyzing the circularity of the system (Figure S19a). In this figure, we compare the effect of changing the diffusion coefficient of species B in a reactive simulation where Q = 10 μL/min. Results show that when B diffuses slower, the system is not effectively stabilized, and the circularity drops faster. Once the instability grows, the reaction effects increase the circularity. However, once the displacing solution reaches the border of the domain, the total displacement is less effective. When B diffuses faster, the combination of reactive and diffusive processes produces an effective stabilization of the system that increases the effective displacement. This can be observed in figure S19b when both situations are compared at the same time. The effect produced by the fast diffusion of species B is fundamental for obtaining a stable displacement when the reaction occurs. This demonstrates that not only the reaction plays a major role in the pattern stabilization/formation, but also diffusion. More specifically, the diffusion effects are fundamental in the pattern stabilization mechanism.

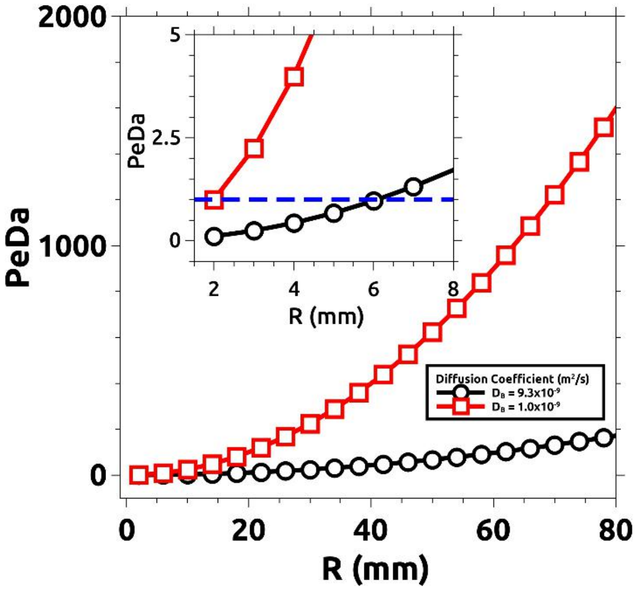

Figure S18: PeDa number estimation considering that B diffuses slower ($1.0x10^{-9}$ m^2/s) and faster ($9.3x10^{-9}$ m^2/s). A PeDa number below 1 indicates that diffusion effects are relevant compared to reaction effects. The estimation shows that there is a range of radii where PeDa < 1 if B diffuses faster (This is indicated in the inset of the figure).

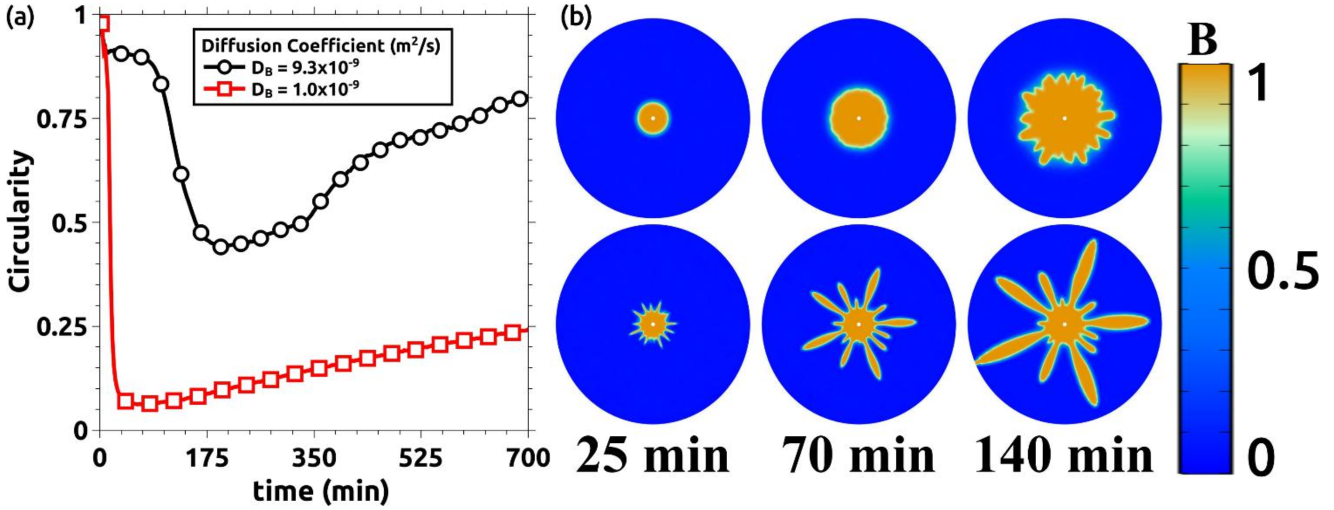

Figure S19: Effect of the diffusion coefficient of species B for (a) the circularity and (b) the pattern formation. In (a) the circularity is compared for the two cases presented in Figure S18. As can be seen, the circularity shows larger values (this is, more stable situation), when B diffuses faster. (b) The effect of diffusion of B is particularly noticeable at the beginning of the simulation where two simulations are compared keeping the remaining parameters as indicated in Table 1. The faster diffusion combined with the reactivity produces the stabilization of the system that improving the total displacement process. In both cases Q = 10 μL/min.

12.- Mesh independence study.

A mesh independence study is necessary to ensure the validity of the numerical results. Figure S20 shows in a semi-log plot the effect on the circularity produced by the mesh resolution in a representative simulation of the direct experiment (Q = 5 µL/min). All measurements are taken at t/t_f =0.5 As can be seen, the circularity is strongly affected by a low mesh resolution. We consider that the results are mesh independent when the circularity differs less than 3% between consecutive refinements. We performed all the simulations by using the coarsest mesh that satisfied such a condition.

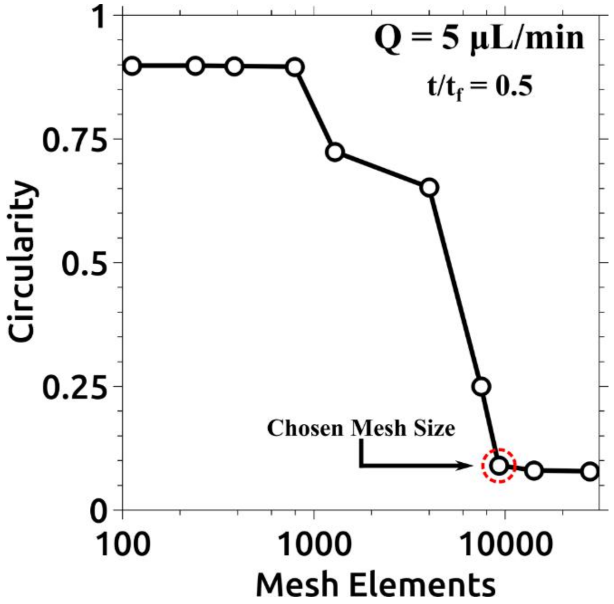

Figure S20: Mesh independence study done by studying the circularity variation for different mesh sizes in a representative case of the direct experiment (Q = 5 µL/min). All values were obtained at normalized time t/t_f = 0.5. Our choice of the mesh size was done after verifying that the difference in circularity between two consecutive simulations is less than 3%. In our case, this value was obtained for approximately 10000 mesh elements.

13.- Description of the supplementary movies.

The supplementary movies included are divided into two groups. The first group deals with the direct experiments and simulations where the more viscous solution displaces the less viscous one. The second set of movies includes results from the reverse experiments and simulations, i.e., the less viscous fluid displacing the more viscous one.

For the direct experiments we present:

1_DirectReactOpticQ=2.5uLmin.avi: presents a typical experiment where the instability is induced by the chemical reaction at the interface with Q=2.5 µL/min

2_DirectReactOpticQ=2500uLmin.avi: same experiment with a much larger flow rate, Q=2500 µL/min. In this case, the interfacial chemical reaction does not induce interface instability.

3_DirectReactSchlieQ=3ulmin.avi: detail of the chemically induced instability at the interface recorded using Schlieren technique. Here, the details of the precipitation process crucial in understanding the mechanism are clearly observed. Q=3 μL/min

4_DirectControlOpticQ=5uLmin.avi: control experiment without reaction at the interface (Q=5 μL/min). In this case, no instability is observed clearly demonstrating that the instability observed in the previous movies is induced by the chemical reaction at the interface.

5_DirecExperiment_SimulationsQ=0.5-1-5-200uLmin.avi: simulation results of the direct system for several flow rates (Q = 0.5, 1, 5, and 200 μL/min), where the concentration fields of species A and C are shown overlayered.

For the reverse experiments:

11_ReverseReactOpticQ=10uLmin.avi: typical experiment at a low flow rate with an interfacial chemical reaction (Q=10 μL/min). There is no fluid instability in this experiment and the more viscous solution (in blue) is mostly displaced by the less viscous one.

12_ReverseReactOpticQ=200uLmin.avi: same experiment with a much larger flow rate, Q=200 μL/min. In this case, the interfacial chemical reaction does not prevent an instability to occur and the less viscous fluid does not displace effectively the more viscous solution in blue.

13_ReverseReactSchlieQ=5uLmin_Schlieren.avi: detail of the chemically induced instability deactivation at the interface recorded using Schlieren technique. Here, the details of the precipitation process crucial in understanding the mechanism are clearly observed. Q=5 μL/min

14_ReverseControlOpticQ=10uLmin.avi: control experiment without reaction at the interface (Q=10 μL/min). In this case, the configuration is unstable, and a fingering instability is immediately induced at the interface.

15_ReverseExperiment_SimulationQ=10-200uLmin.avi: comparison between reactive and non-reactive simulations for Q=10 and 200 μL/min.

REFERENCES

1. Settles, G. S. *Schlieren and Shadowgraph Techniques*. *Schlieren and Shadowgraph Techniques* (Springer-Verlag, Berlin, 2001). doi:10.1007/978-3-642-56640-0.
2. Molnár, I., Takács, N., Kurin-Csörgei, K., Orbán, M. & Szalai, I. Some general features in the autocatalytic reaction between sulfite ion and different oxidants. *Int. J. Chem. Kinet.* **45**, 462–468 (2013).
3. Szalai, I. & De Kepper, P. Pattern formation in the ferrocyanide-iodate-sulfite reaction: The control of space scale separation. *Chaos* **18**, (2008).
4. Zeebe, R. E. & Wolf-Gladrow, D. A. *CO_2 in seawater: equilibrium, kinetics, isotopes*. *Elsevier Oceanography Series* (2001).
5. Betts, R. H. & Voss, R. H. The kinetics of oxygen exchange between the sulfite ion and water. *Can. J. Chem.* **48**, 2035–2041 (1970).
6. Terao, K. Poly (acrylic acid)(PAA). *Encycl. Polym. Nanomater.* 1654–1658 (2015).
7. Adamczyk, Z., Bratek, A., Jachimska, B., Jasiński, T. & Warszyński, P. Structure of poly(acrylic acid) in electrolyte solutions determined from simulations and viscosity measurements. *J. Phys. Chem. B* **110**,

22426–22435 (2006).

8. Kobayashi, S. & Müllen, K. *Encyclopedia of Polymeric Nanomaterials-With 2021 Figures and 146 Tables.* (Springer, 2015).

9. Escala, D. M. & Muñuzuri, A. P. Interface Fingering Instability Triggered by a Density-Coupled Oscillatory Chemical Reaction via Precipitation. *Langmuir* **35**, 13769–13781 (2019).

10. Shukla, P. & De Wit, A. Fingering dynamics driven by a precipitation reaction: Nonlinear simulations. *Phys. Rev. E* **93**, 023103 (2016).

11. Brau, F., Schuszter, G. & De Wit, A. Flow Control of A+B → C Fronts by Radial Injection. *Phys. Rev. Lett.* **118**, 134101 (2017).

12. Escala, D. M., De Wit, A., Carballido-Landeira, J. & Munuzuri, A. P. Viscous Fingering Induced by a pH-Sensitive Clock Reaction. *Langmuir* **35**, 4182–4188 (2019).

13. Escala, D. M., Muñuzuri, A. P., De Wit, A. & Carballido-Landeira, J. Temporal viscosity modulations driven by a pH sensitive polymer coupled to a pH-changing chemical reaction. *Phys. Chem. Chem. Phys.* **19**, 11914–11919 (2017).

14. Kovacs, K., McIlwaine, R. E., Scott, S. K. & Taylor, A. F. pH oscillations and bistability in the methylene glycol-sulfite- gluconolactone reaction. *Phys. Chem. Chem. Phys.* **9**, 3711–3716 (2007).

15. Kovacs, K., McIlwaine, R. E., Scott, S. K. & Taylor, A. F. An organic-based pH oscillator. *J. Phys. Chem. A* **111**, 549–551 (2007).

16. Palencia, M. & Rivas, B. L. Adsorption of linear polymers on polyethersulfone membranes: Contribution of divalent counterions on modifying of hydrophilic-lipophilic balance of polyelectrolyte chain. *J. Memb. Sci.* **372**, 355–365 (2011).

17. Agmon, N. The Grotthuss mechanism. *Chem. Phys. Lett.* **244**, 456–462 (1995).

18. Fischer, S. A., Dunlap, B. I. & Gunlycke, D. Correlated dynamics in aqueous proton diffusion. *Chem. Sci.* **9**, 7126–7132 (2018).

19. Nagatsu, Y., Kondo, Y., Kato, Y. & Tada, Y. Effects of moderate Damköhler number on miscible viscous fingering involving viscosity decrease due to a chemical reaction. *J. Fluid Mech.* **625**, 97–124 (2009).

20. Fernandez, J. & Homsy, G. M. Viscous fingering with chemical reaction: Effect of in-situ production of surfactants. *J. Fluid Mech.* 267–281 (2003) doi:10.1017/S0022112002003683.

21. Nagatsu, Y., Matsuda, K., Kato, Y. & Tada, Y. Experimental study on miscible viscous fingering involving viscosity changes induced by variations in chemical species concentrations due to chemical reactions. *J. Fluid Mech.* **571**, 475–493 (2007).

22. Nagatsu, Y., Iguchi, C., Matsuda, K., Kato, Y. & Tada, Y. Miscible viscous fingering involving viscosity changes of the displacing fluid by chemical reactions. *Phys. Fluids* **22**, 1–13 (2010).

23. Nagatsu, Y., Kondo, Y., Kato, Y. & Tada, Y. Miscible viscous fingering involving viscosity increase by a chemical reaction with moderate Damköhler number. *Phys. Fluids* **23**, (2011).
